# METTL1-mediated m^7^G modification of valine tRNAs drives metabolic adaptation in pancreatic ductal adenocarcinoma cells

**DOI:** 10.1172/JCI204312

**Published:** 2026-08-06

**Authors:** Jiabei Zhu, Qi Zhang, Douglas Evans, Rui Su, Qiuhui Pan, Ajay Goel

**Affiliations:** 1 Department of Molecular Diagnostics and Experimental Therapeutics, Beckman Research Institute of City of Hope, Biomedical Research Center, Monrovia, California, USA.; 2 Department of Clinical Laboratory, Shanghai Children’s Medical Center, Shanghai Jiao Tong University School of Medicine, Shanghai, China.; 3 Department of Surgery, Medical College of Wisconsin, Milwaukee, Wisconsin, USA.; 4 Department of Systems Biology, Beckman Research Institute of City of Hope, Monrovia, California, USA.; 5 City of Hope Comprehensive Cancer Center, Duarte, California, USA.

**Keywords:** Gastroenterology, Oncology, Mitochondria, Noncoding RNAs, RNA processing

## Abstract

Transfer RNA (tRNA) modifications play a critical role in regulating codon-specific mRNA translation and enabling tumor cell adaptation. The RNA methyltransferase 1 (METTL1) installs N7-methylguanosine (m^7^G) modifications on tRNAs, thereby shaping codon usage and translational output. However, the function and mechanistic contribution of the METTL1/tRNA axis in pancreatic ductal adenocarcinoma (PDAC) remain poorly defined. Here, we showed that METTL1 was overexpressed in PDAC tissues and that elevated METTL1 expression was associated with poor patient survival. Genetic ablation of METTL1 markedly suppressed PDAC cell proliferation, migration, and tumor growth in vitro and in vivo. Mechanistically, METTL1 loss selectively reduced m^7^G-modified valine tRNAs — particularly Val-AAC, Val-CAC, and Val-TAC — leading to impaired translation of valine-enriched OXPHOS transcripts. As a consequence, METTL1 deficiency disrupted mitochondrial respiration and energy production in PDAC cells. Consistent with this model, valine tRNA levels were elevated in PDAC tissues, and their selective depletion phenocopied METTL1 loss by impairing mitochondrial bioenergetics and tumor cell fitness. Thus, the METTL1/valine tRNA axis promoted PDAC progression through codon-dependent translational control of the mitochondrial electron transport chain and oxidative metabolism. Together, our findings identify a METTL1/tRNA/mitochondria signaling axis as a metabolic vulnerability and a promising therapeutic target in pancreatic cancer.

## Abstract 2

N/A

## Graphical abstract

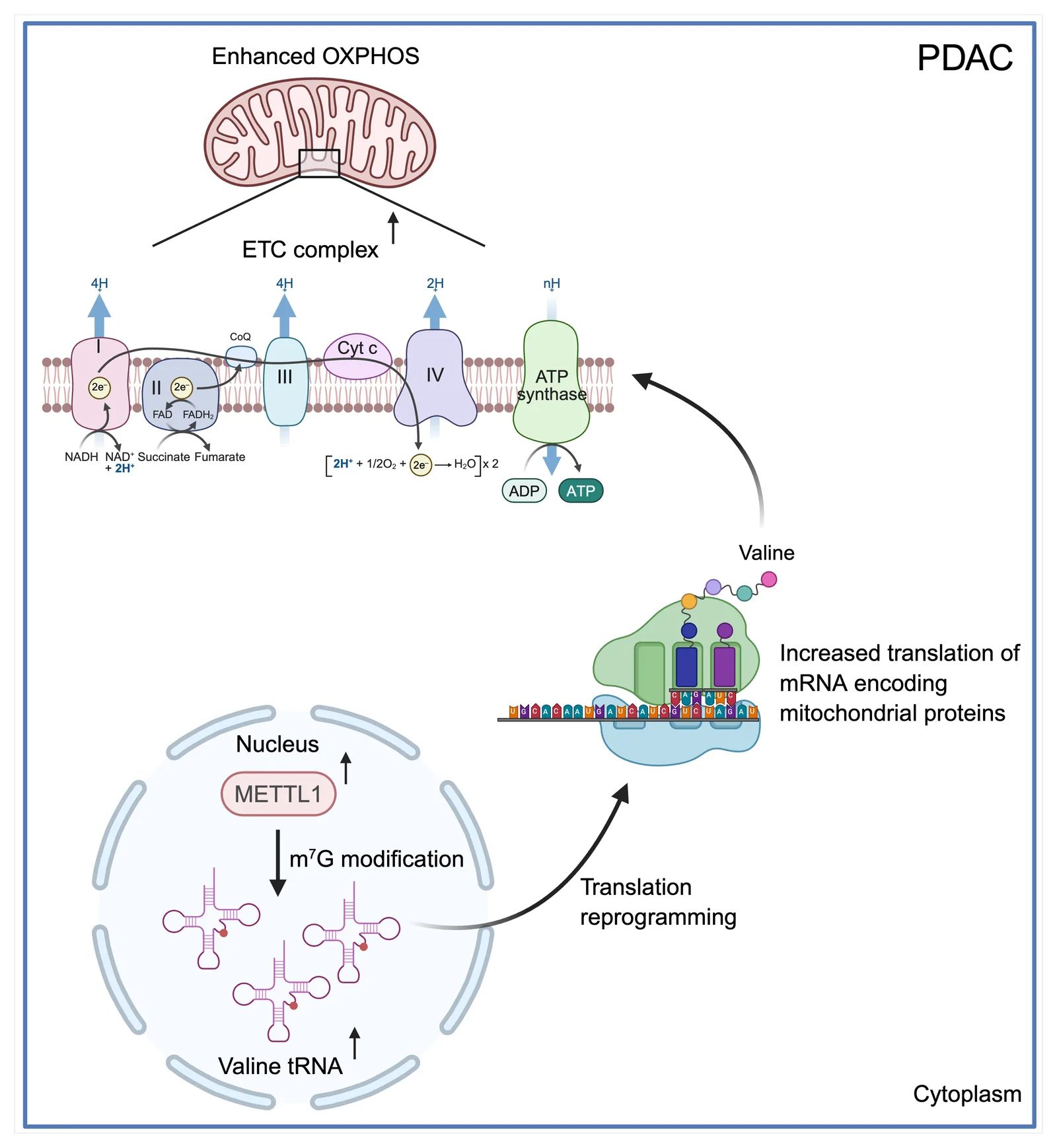

## Introduction

Pancreatic ductal adenocarcinoma (PDAC) is the most common malignancy of the pancreas and remains one of the deadliest human cancers, with a 5-year survival rate below 10% (1). PDAC has surpassed breast cancer to become the third leading cause of cancer-related death in the United States (2) and is projected to become the second leading cause, after lung cancer, by 2040 (3). Surgical resection combined with systemic chemotherapy, with or without radiation, remains the only potentially curative treatment for PDAC. However, despite incremental advances in systemic therapies, long-term survival outcomes remain dismal. This poor prognosis largely reflects the fact that approximately 90% of patients present with advanced disease, and more than half have systemic metastases at initial diagnosis (4). The steadily increasing incidence of pancreatic cancer underscores an urgent need to identify novel therapeutic vulnerabilities that can benefit the majority of patients (5).

PDAC is driven by a complex landscape of genetic and epigenetic alterations that govern tumor initiation, progression, and therapeutic resistance. While recurrent driver mutations in KRAS, TP53, CDKN2A, and SMAD4 define early tumorigenesis, substantial inter-patient genetic heterogeneity gives rise to numerous less-frequent mutations that further promote disease progression (6, 7). Intriguingly, PDAC exhibits relatively low overall mutational burden compared with other solid malignancies, suggesting that epigenetic and posttranscriptional regulatory mechanisms play a prominent role in shaping disease biology (8). Recent studies of the ‘‘epitranscriptome’’ have revealed important roles of RNA modifications in PDAC pathogenesis (9).

Among these, N7-methylguanosine (m^7^G) at transfer RNA (tRNA) nucleotide position 46 is one of the most abundant and conserved, stabilizing rRNA tertiary structure and ensuring translational fidelity (10). This modification is catalyzed by a heterodimeric methyltransferase complex composed of methyltransferase 1 (METTL1) and WD repeat domain 4 (WDR4) (11). Cellular tRNA abundance and modification states are tightly regulated to match mRNA codon usage and optimize protein synthesis (12). Emerging evidence indicates that translational reprogramming is a key driver of oncogenic phenotypes (13). Although dysregulated translation initiation has long been recognized as a hallmark of cancer, recent studies highlight altered tRNA availability and codon usage bias as an additional regulatory layer controlling cancer-specific translation programs (14). Indeed, tRNA hypomodification can selectively influence gene expression and affect tumor growth, progression, and metastasis (15). METTL1 overexpression has been reported in multiple cancer types and is associated with poor clinical outcomes (16–19). However, how m^7^G-modified tRNAs shape the translational landscape and contribute to PDAC remains largely unexplored.

In this study, we identify an oncogenic translational program in PDAC orchestrated by a METTL1/valine tRNA axis. We show that METTL1 was overexpressed in PDAC tissues and that its elevated expression strongly correlated with an adverse patient prognosis. METTL1 depletion reduced m^7^G modification of valine tRNAs — particularly Val-AAC, Val-CAC, and Val-TAC — resulting in impaired PDAC cell proliferation and migration. Mechanistically, loss of METTL1-dependent valine tRNA modification selectively suppressed the translation of valine-enriched mRNAs encoding components of oxidative phosphorylation (OXPHOS), thereby compromising mitochondrial bioenergetics. Consistent with this model, valine tRNA levels were elevated in PDAC tissues, and targeted knockdown (KD) of these tRNAs phenocopies the effects of METTL1 depletion. Together, our findings demonstrate that METTL1-driven valine tRNA modification promotes PDAC progression by remodeling the translational landscape to support metabolic adaptation. This work uncovers a role for the METTL1/tRNA axis in mitochondrial metabolism and highlights a promising therapeutic vulnerability in pancreatic cancer.

## Results

### METTL1 is amplified and overexpressed in PDAC tissues.

m^7^G modification is catalyzed by a heterodimeric methyltransferase complex comprising METTL1 and WDR4, in which METTL1 serves as the catalytic subunit harboring the S-adenosylmethionine–binding (SAM-binding) domain responsible for m^7^G methyl group transfer at position 46 of the target tRNAs, while WDR4 functions as a structural cofactor that stabilizes METTL1 and facilitates substrate tRNA recognition (11). METTL1 resides within the 12q13–14 chromosomal region, which is frequently amplified across multiple cancer types (20). Examination of The Cancer Genome Atlas (TCGA) showed that METTL1 is amplified in 11.73% of pancreatic cancer patients and is commonly amplified in other tumor types (Figure 1A). To assess the potential role of METTL1 in pancreatic cancer, we first examined its expression using publicly available transcriptomic datasets, including PDAC tissues from TCGA database and normal pancreatic tissues from the Genotype Tissue Expression (GTEx) database. We observed that METTL1 expression was markedly higher in PDAC tissues than in normal pancreatic tissues (Figure 1B, *P* < 0.001). Consistent with elevated METTL1 expression, WDR4 expression was also upregulated in PDAC tissues (Figure 1C, *P* < 0.001). In addition, Kaplan-Meier analysis revealed that patients with higher METTL1 or WDR4 expression had worse disease-free survival (DFS) (Figure 1D, *P* = 0.0398; Figure 1E, *P* = 0.0256). Subsequently, we performed quantitative reverse transcription PCR (qRT-PCR) to detect mRNA expression of METTL1 and WDR4 in an independent cohort of 40 paired PDAC and adjacent nontumor (AN) tissue specimens from the Medical College of Wisconsin (Milwaukee, Wisconsin, USA) (Supplemental Table 1; supplemental material available online with this article; https://doi.org/10.1172/JCI204312DS1). Higher mRNA expression of METTL1 and WDR4 was observed in PDAC tissues compared with the AN tissues (Figure 1, F and G), suggesting a potential dysregulation of m^7^G modification in PDAC pathogenesis. Western blotting further confirmed the increased METTL1 protein expression in patient-derived PDAC tissues compared with AN tissues (Figure 1H). To further examine the protein levels of METTL1 and WDR4, we evaluated IHC staining for protein expression available on the Human Protein Atlas (HPA) portal (https://www.proteinatlas.org/). Consistent with the RNA expression pattern, the staining results revealed increased METTL1 and WDR4 protein expression in the tumor tissues compared with normal pancreatic tissues (Figure 1I). Taken together, these cancer genetics and expression analyses implicate that METTL1 is amplified and aberrantly upregulated in PDAC tissues, suggesting its potential clinical significance in PDAC.

### METTL1 is required for PDAC cell growth, migration, and tumorigenicity.

To investigate the biological role of METTL1 in PDAC, we first assessed its expression in the human pancreatic duct epithelial cell line HPDE6c7 and in several pancreatic cancer cell lines (BxPC-3, capan-2, MiaPaCa-2, and PANC-1) using qRT-PCR and Western blotting. Both mRNA and protein levels of METTL1 were elevated in multiple PDAC cell lines compared with HPDE6c7 cells (Supplemental Figure 1, A and B). Among these, capan-2 and PANC-1 cells showed a modest increase, with the highest expression observed in MiaPaCa-2 and BxPC-3 cells lines. We next knocked out METTL1 in BxPC-3 and MiaPaCa-2 cell lines using CRISPR/Cas9, and we verified successful deletion by Western blotting (Figure 2A). We performed 3-[4,5-dimethylthiazol-2-yl]-2,5 diphenyl tetrazolium bromide (MTT) and colony formation assays to investigate the role of METTL1 in regulating PDAC cell survival. The results showed that METTL1 deletion led to potent inhibition of cell growth (Figure 2, B and C) and a substantial suppression of colony formation (Figure 2D) in BxPC-3 and MiaPaCa-2 cells. Next, we examined the effect of METTL1 KO on PDAC cell migration. The wound-healing assay demonstrated that there were fewer migrated cells across the baseline in the METTL1-KO cells than in control cells after 24 hours (Figure 2E).

To determine whether elevated METTL1 expression is sufficient to promote malignant phenotypes, we induced transient overexpression of WT METTL1 (OE-METTL1^WT^) or a catalytically inactive mutant (OE-METTL1^MUT^) in capan-2 cells. Overexpression of METTL1^WT^ markedly increased cell proliferation (Supplemental Figure 1, C and D) and migratory capacity (Supplemental Figure 1E) compared with empty vector controls (OE-NC), whereas METTL1^MUT^ had no apparent effect. These gain-of-function results indicate that METTL1 promotes PDAC cell proliferation and migration in a methyltransferase activity–dependent manner.

We next tested the requirement for METTL1 in tumor formation in vivo by performing mouse xenograft experiments and measuring tumor growth after s.c. transplantation into nude mice. To summarize, we s.c. injected 1 × 10^7^ MiaPaCa-2 cells into nude mice, either with METTL1-KO (*n* = 5) or a non-targeting control single-guide RNA (sgNC) control (*n* = 5) (Figure 2F). The results showed that both tumor volume and weight in the METTL1-KO group were markedly lower than those in the control group (Figure 2, G and H). IHC assays further confirmed that the percentage of Ki67^+^ nuclei was substantially lower in the METTL1-KO group than in the control group (Figure 2I). Overall, our data show an important role of METTL1 in controlling PDAC cell growth, migration, and oncogenicity.

### METTL1 depletion leads to decreased levels of m^7^G-modified valine tRNAs and translation defects.

To understand the molecular mechanism underlying the cell growth defects in METTL1-KO cells, we measured m^7^G levels in METTL1-KO samples compared with controls using a dot blot assay. We observed a decrease in the overall levels of m^7^G modification in small RNAs (Figure 3, A–C). Next, we detected alterations in the m^7^G-modified tRNA subset after METTL1 depletion in PDAC cells (Figure 3D). We validated a decrease in the abundance of the m^7^G-modified valine tRNAs in METTL1-KO cells compared with control cells, including Val-AAC, Val-CAC, and Val-TAC (Figure 3, E–G). Interestingly, we did not observe a substantial decrease in tRNAi-Met (m^7^G-modified), suggesting that loss of METTL1 may not induce a global defect in translation initiation (Figure 3H). Besides this, we detected no apparent changes in other tRNA species (Supplemental Figure 2, A–O). To test whether METTL1 is the enzyme responsible for valine tRNA m^7^G methylation using an antibody-dependent method, we performed a RNA immunoprecipitation–quantitative PCR (RIP-qRT-PCR) assay in BxPC-3 and MiaPaCa-2 cells and observed that candidate tRNAs had marked enrichment in the METTL1 pull-downs relative to the IgG pull-downs (Figure 3, I and J). To further validate this observation, we measured m^7^G levels in three valine tRNAs from METTL1-KO cells compared with control samples using m^7^G methylated RNA immunoprecipitation (m^7^G-MeRIP) followed by qRT-PCR and observed the decrease in methylation signals of Val-AAC, Val-CAC, and Val-TAC after loss of METTL1 in PDAC cells (Figure 3, K and L). RNA modification can regulate tRNA structure stability and maintain a healthy tRNA pool (21). To investigate the mechanism by which METTL1 regulates valine tRNA expression, we performed RNA decay assays to detect the stability of valine tRNAs. We found that the half-lives of 3 valine tRNAs were markedly reduced in METTL1-KO cells compared with control cells (Figure 3, M–O). To further investigate the role of METTL1’s methyltransferase activity in regulating valine tRNAs, we reintroduced METTL1^WT^ and METTL1^MUT^ into METTL1-KO cells. The results showed that METTL1 deficiency substantially downregulated the expression levels of Val-AAC (Supplemental Figure 3, A and D), Val-CAC (Supplemental Figure 3, B and E), and Val-TAC (Supplemental Figure 3, C and F) in PDAC cells, and overexpression of METTL1^WT^, but not METTL1^MUT^, restored valine tRNA levels, confirming the requirement of METTL1 methyltransferase activity for regulation of tRNAs. Taken together, these data indicate that loss of METTL1 led to reduced m^7^G modification of valine tRNAs and decreased the stability of hypomodified tRNAs.

### Valine tRNAs are upregulated in PDAC and are necessary for cancer cell growth and migration.

Alterations in tRNA expression are presumed to accommodate proteome requirements and to support tumor progression and metastasis (22). To assess the potential role of valine tRNAs in PDAC, we examined the expression of Val-AAC, Val-CAC, and Val-TAC in an independent cohort consisting of 32 pairs of PDAC and AN frozen tissue specimens obtained from the Medical College of Wisconsin, USA. The results revealed an apparent increase in the levels of 3 valine tRNAs in PDAC tissues compared with AN tissues (Figure 4A). Pearson correlation analysis demonstrated a marked positive correlation between METTL1 and Val-AAC (*r* = 0.6296, *P* = 0.0001), Val-CAC (*r* = 0.5535, *P* = 0.0012), and Val-TAC (*r* = 0.7491, *P* < 0.0001) in a clinical cohort of 32 PDAC cases (Figure 4B).

Consistent with these findings in clinical samples, all 3 valine tRNAs were markedly elevated in PDAC cell lines compared with HPDE6c7 cells, with the highest expression observed in BxPC-3 and MiaPaCa-2 cells (Supplemental Figure 3, G–I), further supporting the upregulation of valine tRNAs as a characteristic feature of malignant PDAC. To assess the functional contribution of valine tRNAs to PDAC survival and metastasis, we further generated stable shRNA–mediated KD of valine tRNAs in PDAC cell lines (Figure 4C). Results from MTT and colony formation assays showed that KD of valine tRNAs resulted in potent inhibition of cell growth (Figure 4D) and substantially reduced colony formation (Figure 4E) in BxPC-3 and MiaPaCa-2 cells. Next, we examined the effect of valine tRNAs on PDAC cell migration. The wound-healing assay demonstrated that there were fewer migrated cells across the baseline in the valine tRNA-deficient cells than in control cells after 24 hours (Figure 4F). Taken together, these analyses highlight valine tRNAs as possible mediators of the oncogenic effects of METTL1 in PDAC.

### METTL1 depletion leads to valine codon–biased mRNA translational changes.

Cancer cells selectively promote the translation of specific oncogenic transcripts to facilitate cancer survival and progression (23). Considering the changes in tRNA abundance upon METTL1 depletion, we reasoned that this could affect mRNA translation. Therefore, we first assessed the effect of METTL1 depletion on global mRNA translation and protein synthesis by monitoring puromycin incorporation into nascent peptides in BxPC-3 and MiaPaCa-2 cells. In line with our observation of no apparent changes in tRNAi-Met, METTL1 depletion did not substantially alter global protein synthesis in PDAC cells (Figure 5A). We next considered the possibility that METTL1 loss affects the translation of specific mRNAs that regulate oncogenic transformation. To investigate this possibility, we performed polysome profiling experiments to evaluate codon usage and relative translation efficiencies (TEs) in PDAC cells depleted or not of METTL1 (Figure 5, B and C, and Supplemental Tables 2 and 3). This analysis revealed greater than 1.5-fold changes (FCs) in the TEs of numerous mRNAs in METTL1-KO cells compared with control cells (BxPC-3: 3,776; MiaPaCa-2: 1,314). Among these, 223 mRNAs showed consistently decreased TEs in both BxPC-3 and MiaPaCa-2 METTL1-KO cells (Figure 5D). Gene Ontology (GO) enrichment analysis based on the METTL1-dependent genes with reduced TE identified enrichment of pathways mainly related to translation, including “ribosomal large subunit assembly,” “ribosomal large subunit biogenesis,” “positive regulation of ribosome,” “cytosolic ribosome,” and “mitochondrial ribosome.” In addition, both GO and Kyoto Encyclopedia of Genes and Genomes (KEGG) analyses identified pathways related to OXPHOS, including OXPHOS uncoupler activity and NADH dehydrogenase (ubiquinone) activity (Figure 5, E and F), suggesting that METTL1 can regulate mitochondrial proteostasis.

To determine whether valine codon usage is systematically altered in translationally downregulated genes, we performed codon usage analysis on principal mRNA coding sequences (CDSs) as defined by annotation of principal and alternative splice isoforms (APPRIS) annotations in GENCODE version 49. Codon frequencies were quantified as counts per 1,000 codons, and log_2_ FCs were calculated for TE-down genes relative to TE-up genes (log_2_FC down vs. up) and the genome-wide average (log_2_FC down vs. all). Four-quadrant scatterplot analysis revealed that several m^7^G-modified valine codons are consistently enriched in TE-down genes compared with both TE-up genes and the global average (Figure 5G and Supplemental Table 4). Consistently, the combined usage of m^7^G-modified valine codons (GTT + GTA + GTG) was markedly higher in TE-down genes compared with all other genes (Figure 5H). Specifically, GTA and GTT codons were markedly enriched in mRNAs with decreased TE following METTL1-KO (Figure 5, I and J), whereas GTG codon usage showed only a modest change (Figure 5K). This likely reflects the fact that genome-wide averages may underestimate the translational effect on specific subsets of GTG-enriched transcripts. Importantly, GTC — the only valine codon not subject to METTL1-dependent m^7^G modification — showed no enrichment in TE-down genes, indicating that the observed enrichment pattern was specific to m^7^G-modified valine codons rather than a general increase in valine codon content (Figure 5L). These findings further support our observation that genes enriched for m^7^G-modified codons were preferentially translationally downregulated in METTL1-KO cells. In particular, Val-AAC and Val-TAC, which decode GTT and GTA, were responsible for some of the changes in protein synthesis and mitochondrial energetics upon METTL1-KO.

### METTL1/valine tRNA depletion leads to altered mitochondrial bioenergetics in PDAC cells.

OXPHOS, which occurs in the inner mitochondrial membrane, is responsible for generating ATP in mitochondria. Mitochondrial abnormalities lead to altered bioenergetics and tumor pathogenesis. Recent studies have shown that mitochondrial proteins are generally enriched for valine codons, and several of these proteins are translationally downregulated under valine-restricted conditions (24). Function enrichment analyses of our polysome profiling data revealed that METTL1-dependent genes with decreased TE were enriched in OXPHOS-related processes. Next, we performed ribosome fractionation to analyze the distribution of these OXPHOS-associated mRNAs in the untranslated and translated pools. The results showed that mRNAs encoding ATP5H, NDUFC1, and NDUFV2, core subunits of the electron transport chain (ETC), exhibited substantially reduced translation in METTL1-KO cells (Figure 6, A–C). In addition, mRNAs encoding SLC25A4 and UCP2, key regulators of OXPHOS uncoupling, also displayed markedly reduced translation upon METTL1 loss (Figure 6, D and E).

To further investigate whether the redistribution of these OXPHOS-related transcripts across polysome fractions reflects codon-specific translational regulation, we performed systematic analyses of m^7^G-modified valine codon (GTT + GTA + GTG) usage across the OXPHOS-related genes in the TE-down group and found a collectively higher mean GTT + GTA + GTG frequency compared with the global mean of 46.7 of 1,000 codons (Supplemental Figure 4, A and B). Notably, NDUFV2 harbored a GTT + GTA + GTG frequency of 64.3 of 1,000 codons, exceeding the global mean, whereas its GTC frequency (8.0 of 1,000) falls below the global average (13.7 of 1,000), providing an internal codon-type control within the same transcript. Similarly, UCP2 displayed a GTT + GTA + GTG frequency of 55.0 of 1,000 codons, also above the global mean. Importantly, individual OXPHOS transcripts showed differential enrichment of specific m^7^G-modified valine codons relative to the global average, such that distinct subsets of OXPHOS genes were preferentially sensitive to the loss of each cognate tRNA. This heterogeneous codon composition provides a mechanistic basis for the coordinated yet codon-specific translational downregulation of mitochondrial targets upon METTL1 depletion. By contrast, the non-m^7^G GTC codon was not enriched across OXPHOS genes as a group (mean 12.5 of 1,000 vs. global 13.7 of 1,000), confirming that the enrichment was specific to codons decoded by METTL1-dependent m^7^G-modified tRNAs rather than reflecting a general increase in overall valine codon abundance. Consistently, Western blotting confirmed reduced protein levels of NDUFV2 and UCP2 after depletion of METTL1 or valine tRNAs in PDAC cells (Figure 6, F and G). Importantly, protein levels of CD44 and calnexin — two low-valine-codon proteins not expected to be translationally sensitive to METTL1 loss, consistent with the polysome profiling data — were unchanged upon METTL1 depletion (Supplemental Figure 4, C and D), confirming that METTL1 depletion selectively impaired translation of valine codon–enriched transcripts rather than globally suppressing protein synthesis.

To characterize the functional consequences of reduced translation of mRNAs encoding OXPHOS proteins, we performed Seahorse Mito Stress testing to assess mitochondrial bioenergetics following METTL1 depletion. We observed that loss of METTL1 reduced basal and maximal respiration in BxPC-3 (Figure 6H) and MiaPaCa-2 (Figure 6I) cells, suggesting that METTL1 depletion impaired OXPHOS in PDAC cells. To determine whether altered valine tRNAs contribute to METTL1-mediated defects in respiratory activity, we assessed mitochondrial respiration function after KD of valine tRNAs in PDAC cells. Consistent with METTL1 loss phenotypes, KD of valine tRNAs also reduced basal and maximal respiration in BxPC-3 (Figure 6J) and MiaPaCa-2 (Figure 6K) cells. To determine whether the observed respiratory impairment reflected a functional deficit or loss of mitochondrial mass, we assessed mitochondrial content in METTL1-KO cells. Western blotting analysis of the mitochondrially encoded proteins MT-ND1 and MT-CO2 in METTL1-KO cells showed no reduction relative to control cells (Supplemental Figure 4, C and D). Consistently, qRT-PCR analysis of mitochondrial DNA (mtDNA) copy numbers showed no apparent difference in the MT-ND1/β2M ratio between METTL1-KO and control cells (Supplemental Figure 4E). Together, these findings indicate that METTL1 depletion impaired mitochondrial respiratory function without reducing mitochondrial mass or biogenesis, indicating a functional rather than structural mechanism. To further assess the clinical relevance of the METTL1/tRNA/OXPHOS axis, we measured NADH:ubiquinone oxidoreductase core subunit V2 (NDUFV2) and uncoupling protein 2 (UCP2) protein levels in 10 paired PDAC and AN tissues. Both proteins were consistently elevated in tumors compared with matched AN tissues across most patient tissue pairs (Supplemental Figure 4F), indicating translational upregulation of OXPHOS-related targets downstream of METTL1 in PDAC. Collectively, METTL1/valine tRNAs enhanced OXPHOS and energy production by affecting the translation of OXPHOS mRNAs in PDAC cells.

### Overexpression of valine tRNAs and OXPHOS protein recapitulates METTL1-mediated oncogenic changes.

To explore the role of METTL1-regulated valine tRNAs in PDAC oncogenic transformation, we first conducted rescue assays by inducing overexpression of individual valine tRNAs in METTL1-KO cells. Ectopic expression of Val-AAC (Supplemental Figure 5, A–D) or Val-CAC (Supplemental Figure 5, E–H) partially restored PDAC cell proliferation and colony formation following METTL1 depletion, demonstrating that both tRNA species individually contributed to the METTL1-driven oncogenic program. Notably, the partial but apparent rescue achieved by Val-CAC alone provides direct functional evidence for its indispensable role in METTL1-dependent oncogenicity, consistent with the selective enrichment of GTG codons in key OXPHOS transcripts and explaining why even a modest alteration in GTG frequency in the TE-down group translated into meaningful functional consequences for these specific targets. Nevertheless, the incomplete rescue by any single valine tRNA indicates that no individual tRNA species is sufficient to fully compensate for METTL1 loss, suggesting a cooperative requirement for the complete complement of m^7^G-modified valine tRNAs. To test this hypothesis, we next overexpressed all 3 valine tRNAs simultaneously in METTL1-KO cells (Figure 7, A and B). Combined ectopic expression of Val-AAC, Val-CAC, and Val-TAC fully restored the cell growth (Figure 7, C and D), colony formation (Figure 7E), and migratory capacity (Figure 7F) inhibited by KO of METTL1, confirming that the cooperative action of the complete set of m^7^G-modified valine tRNAs was both necessary and sufficient to recapitulate the oncogenic effects of METTL1 in PDAC cells.

To determine whether valine tRNA overexpression also restores downstream OXPHOS protein levels, we assessed NDUFV2 and UCP2 expression in these rescue conditions. Consistent with the restoration of oncogenic phenotypes, overexpression of valine tRNAs restored NDUFV2 and UCP2 protein levels in METTL1-KO cells (Supplemental Figure 4G), further supporting the mechanistic link between the METTL1/valine tRNA axis and the translational control of mitochondrial OXPHOS components. To further confirm whether restoring OXPHOS function downstream of METTL1 is sufficient to rescue proliferative and migratory defects, we induced overexpression of NDUFV2 — a core complex I subunit whose translation is impaired upon METTL1 loss — in METTL1-KO cells. MTT and colony formation assays showed that NDUFV2 overexpression markedly restored proliferation in sgMETTL1 BxPC-3 and MiaPaCa-2 cells (Supplemental Figure 6, A–C). Wound-healing assays further showed that NDUFV2 overexpression markedly rescued the migration deficit in these cells (Supplemental Figure 6D). Collectively, these findings suggest that the METTL1/valine tRNA axis drives oncogenic phenotypes in PDAC cells by promoting the translation of OXPHOS proteins and enhancing cellular energy production.

## Discussion

Here, we identify METTL1 as a potent oncogene that was frequently amplified and upregulated in PDAC. Remarkably, we furthermore found that a cluster of m^7^G-modified valine tRNAs — Val-AAC, Val-CAC, and Val-TAC — was largely responsible for METTL1-driven oncogenicity. Deletion of METTL1 led to a substantial decrease in the levels of valine tRNAs that harbored the m^7^G modification, severely impaired mitochondrial OXPHOS and energy production, and suppressed PDAC cell proliferation and migration, as well as in vivo tumor growth, thereby highlighting METTL1 as a promising therapeutic target in PDAC. Overexpression of WT methyltransferase METTL1, but not a catalytic dead mutant, restored the abundance of these valine tRNAs. Moreover, METTL1 depletion did not result in a global change in translation but rather affected a relatively small subset of mRNAs. Specific reprogramming of mRNA translation modulates phenotype switching and cancer progression with no apparent changes in global mRNA translation (25). In our study, polysome profiling revealed that METTL1 depletion altered the translation of genes involved in OXPHOS, which were enriched in valine codons. Furthermore, KD of m^7^G-modified valine tRNAs phenocopied METTL1 depletion in PDAC cells. Overall, these findings uncover the molecular mechanism of METTL1 oncogenicity, whereby m^7^G-modified valine tRNAs selectively enhance the translation of valine-enriched metabolic regulatory genes, driving PDAC progression.

tRNAs promote stability and translation of transcripts enriched for their codons (26). Altered tRNA expression has been generally regarded as a natural consequence of the high proliferative and metabolic status of cancer cells; however, it is becoming increasingly evident that tRNA dysregulation can play more active roles in tumorigenesis due to the protein synthesis errors, which have been correlated to accelerated tumor growth kinetics (27, 28). Moreover, it is emerging that the relative expression of individual tRNAs varies widely across cancers. Overexpression of tRNAi-Met promotes melanoma metastasis, but this increased metastatic potential does not affect primary tumor proliferation and growth (29). Increased expression of a single tRNA, Arg-TCT-4-1, can promote acute myeloid leukemia initiation in a manner dependent on its AGA decoding function (15). Upregulated valine tRNA biogenesis has been shown to indicate increased demand for valine in protein synthesis in leukemia (24). Our findings show that METTL1-mediated changes in valine tRNA abundance can modulate protein expression in PDAC cells and that cancer cells can evolve to fine-tune the expression of multiple genes to promote metabolic adaptation by modulating valine tRNA levels.

Tumor cell–intrinsic genetic mutations and epigenetic dysregulation can profoundly reshape the tumor microenvironment and metabolism. In addition to mutations in oncogenes and tumor suppressor genes, PDAC progression is strongly driven by alterations in epigenetic regulators, which contribute to highly dynamic phenotypic plasticity, metabolic reprogramming, and remodeling of the tumor microenvironment (30, 31). A recent study has found that tumor-intrinsic loss of the histone H3K36 trimethyltransferase SETD2 shapes heterogeneity in cancer-associated fibroblasts and promotes tumor progression by fueling mitochondrial OXPHOS in PDAC cells via the ABCA8a transporter (32). Our study revealed a highly selective translational vulnerability in which m^7^G-modified valine tRNAs functioned as an important translational checkpoint, conferring metabolic advantages to PDAC cells. The METTL1/valine tRNA axis enhances mitochondrial respiration and drives PDAC progression by selectively promoting the translation of valine-rich OXPHOS genes, including critical regulators of mitochondrial complex I. These findings establish a direct molecular link between tRNA epitranscriptomics and metabolic reprogramming, revealing a layer of translational metabolic regulation.

Taken together, we conclude that valine tRNAs are the dominant and functionally indispensable substrates of METTL1 in PDAC, thereby redefining its substrate specificity in cancer. METTL1-mediated oncogenic effects are due to the altered m^7^G modification and abundance of specific valine tRNAs, in particular Val-AAC/CAC/TAC, which leads to a remodeling of the mRNA ‘‘translatome’’ with selectively increased translation of metabolic regulators enriched in the respective GTT/GTG/GTA codon, thereby enhancing mitochondrial bioenergetics in PDAC. This study highlights the potential of the METTL1/tRNA/mitochondrial axis as a metabolic vulnerability and a promising therapeutic target in pancreatic cancer.

## Methods

### Sex as a biological variable.

Our study examined male and female animals, and similar findings are reported for both sexes.

### Primary patient samples.

Forty pairs of tumor tissues and AN pancreatic tissues were collected from patients with PDAC at Medical College of Wisconsin. The diagnosis of PDAC was established according to the WHO classification. The resected samples were staged according to the International Union Against Cancer (UICC) Tumor-Node-Metastasis (TNM) (sixth edition) classification.

### Xenograft models.

Four-week-old Nu/J nude mice (both sexes) were obtained from The Jackson Laboratory. A total of 1 × 10^7^ MiaPaCa-2 cells with KO of METTL1 (*n* = 5) or an sgNC control (*n* = 5) were s.c. injected into the right flank of each mouse. Tumor size and body weight of the mice were measured every other day. Approximately 35 days after injection, the mice were euthanized, and each tumor was carefully dissected, weighed, and photographed. All experiments involving xenografted mice were conducted in strict accordance with the guidelines outlined in the *Guide for the Care and Use of Laboratory Animals* published by the NIH (National Academies Press, 2011).

### qRT-PCR.

Total RNA was extracted from frozen tissues or cell lysates using the QIAzol Lysis Reagent (Qiagen) and then reverse transcribed to cDNA using a high-capacity cDNA Reverse Transcription Kit (Thermo Fisher Scientific). qRT-PCR was performed using a SensiFAST SYBR Lo-ROX Kit (Bioline) on the QuantStudio 6 Flex Real-Time PCR System (Applied Biosystems). Relative expression was calculated using the 2^−ΔΔCt^ method. β-Actin and 28S rRNA were used as internal controls to normalize gene expression.

For mtDNA copy number analysis, total DNA was extracted from cell lysates using the QIAamp DNA Micro Kit (Qiagen, 56304). Relative mtDNA content was determined by calculating the ratio of the mtDNA copy number (MT-ND1) to the nuclear DNA copy number (β-2-microglobulin [β2M]). The primers used in this study are listed in Supplemental Table 5.

### Cell culture and treatment.

The human PDAC cell lines BxPC-3, capan-2, MiaPaCa-2, and PANC-1 were obtained from the American Type Culture Collection (ATCC). The human pancreatic duct epithelial cell line HPDE6c7 was obtained from Merck Millipore. BxPC-3 cells were cultured in RPMI 1640 Medium (Gibco, Thermo Fisher Scientific, 22400105) supplemented with 10% FBS and 1% penicillin-streptomycin. PANC-1 and MiaPaCa-2 cells were maintained in DMEM (Gibco, Thermo Fisher Scientific; 11965092), whereas capan-2 cells were cultured in McCoy’s 5A Modified Medium (Gibco, Thermo Fisher Scientific, 16600082). HPDE6c7 cells were cultured in Keratinocyte Serum-Free Medium (MilliporeSigma, C-20211) supplemented with epidermal growth factor (MilliporeSigma, GF316) and bovine pituitary extract (MilliporeSigma, 02-103). Cells were incubated at 37°C in a 5% CO_2_ incubator. For RNA decay assays, cells were treated with 5 μg/mL actinomycin D (Gibco, Thermo Fisher Scientific, 11805017) and harvested at 0, 30, 60, and 120 minutes.

### Construction of CRISPR/Cas9-based METTL1-KO PDAC cell lines.

The sgRNAs targeting METTL1 were designed and purchased from Applied Biological Materials. Lentiviruses for KO of METTL1 were generated by cotransfecting the second-generation lentiviral packaging vectors (2.25 μg psPAX2 and 0.75 μg pMD2.G), 3 μg lentiCas9-Blast plasmid (Addgene, 52962), or 3 μg lentiGuide-hygro sgRNA into HEK293T cells using X-tremeGENE HP DNA Transfection Reagent (MilliporeSigma, 6366546001). The lentiviruses were harvested 48 hours and 72 hours after transfection. Cas9 lentivirus particles were added to BxPC-3 and MiaPaCa-2 cells in the presence of 8 μg/mL polybrene (MilliporeSigma, H9268). After 2 rounds of infection, the Cas9 single clones were selected with 20 μg/mL blasticidin (InvivoGen, ant-bl-1) and allowed to grow for 3–4 weeks. The single clones were then infected with sgRNA-expressing lentivirus and selected with 100 μg/mL hygromycin (InvivoGen, ant-hg-5). The sequences of these sgRNAs are listed in Supplemental Table 6, and KO efficiencies were validated by Western blotting.

### Transfection of plasmid.

shRNAs targeting Val-AAC, Val-CAC, and Val-TAC were purchased from Integrated DNA Technologies. The NDUFV2 expression vector (pcDNA3.1/NDUFV2/myc-His) was obtained from Addgene (127491). To construct tRNA expression vectors for Val-AAC, Val-CAC, and Val-TAC, the U6 promoter in the pLKO.1-TRC cloning vector (Addgene, 10878) was first removed by site-directed mutagenesis to generate a ΔU6 backbone (pLKO.1-puro-ΔU6). Each tRNA gene, together with approximately 300 nt of the upstream and 100 nt of the downstream genomic flanking sequences, was PCR amplified from human genomic DNA and cloned into the modified vector, as previously described (15). Plasmid transfections were performed using Lipofectamine 2000 (Invitrogen, Thermo Fisher Scientific, 11668019) according to the manufacturer’s instructions. Primer and shRNA sequences used in this study are listed in Supplemental Table 6.

### Cell proliferation assays.

For cell viability assays, PDAC cells were seeded in 96-well plates at a density of 1,000 cells per well. At the indicated time points, the culture medium was removed, and 100 μL fresh medium containing 10 μL of 5 mg/mL MTT reagent was added to each well. After a 4-hour incubation at 37°C, the MTT reagent was then removed, and 100 μL DMSO was added to each well to dissolve the formazan crystals. Absorbance was measured at 535 nm using a plate reader (Molecular Devices).

For the colony formation assays, 1,000 cells were plated in 12-well plates and cultured for 7–10 days. The cells were then fixed with 4% paraformaldehyde and stained with 0.1% crystal violet (Thermo Fisher Scientific). Colonies were photographed and quantified using ImageJ software (NIH). Data were obtained from at least 3 independent experiments.

### Wound-healing assay.

Cells (5 × 10^5^) were seeded in 6-well plates and incubated at 37°C with 5% CO^2^ for 24 hours. After removing the medium, a scratch was made across the cell monolayer using a 10 μL pipette tip. Cells were gently washed twice with PBS, and 1 mL RPMI-1640 medium was added. Images of the wound area were captured at 0 hours and 24 hours. The migration distance was measured, and results were expressed as a migration index (the migration distance of cells in the experimental group relative to that of the control group).

### Western blotting.

Cells were lysed in RIPA lysis buffer (Thermo Fisher Scientific) with a protease inhibitor cocktail (Thermo Fisher Scientific) on ice for 10 minutes. Protein samples were separated on 10% SDS-PAGE gels and transferred onto nitrocellulose membranes (Cytiva) using a wet transfer method. Membranes were blocked with 5% BSA (MilliporeSigma) for 1 hour at room temperature. After blocking, membranes were incubated overnight at 4°C with primary antibodies, then incubated with HRP-conjugated secondary antibodies (Cell Signaling Technology, no. 7074 or 7076) for 1 hour at room temperature. The immunoblots were subsequently exposed to an HRP-based chemiluminescence kit (Thermo Fisher Scientific), and the signal was imaged using an ECL device (Bio-Rad). The primary antibodies used in this study included anti–β-actin (Proteintech, 66009), anti-METTL1 (Proteintech, 14994-1-AP), and anti-puromycin (Abcam, 315887), CD44 (Cell Signaling Technology, 3570), calnexin (Cell Signaling Technology, 2679), MT-ND1(Proteintech, 19703-1-AP), and MT-CO2 (Proteintech, 55070-1-AP).

### SUnSET assay.

Protein synthesis was assessed using the surface sensing of translation (SUnSET) assay as previously described (33). Briefly, cells were seeded at a density of 1 × 10^6^ cells per well in a 6-well plate and incubated with growth medium supplemented with 20 μg/mL puromycin for the indicated duration. Protein lysates were collected, and nascent protein synthesis was detected using Western blotting with an anti-puromycin antibody.

### m^7^G dot blot assay.

The RNA m^7^G dot blot was performed as previously described with minor modifications (34). Small RNAs were enriched from total RNA using the RNA Clean & Concentrator Kit (Zymo Research, R1015). The indicated amount of small RNAs was denatured in RNA incubation buffer (65.7% formamide, 7.77% formaldehyde, and ×1.33 MOPS) at 65°C for 5 minutes, then chilled on ice. An equal volume of ×20 SSC buffer was added to the samples, and the mixture was thoroughly mixed. The RNA samples were spotted onto an Amersham Hybond-N^+^ membrane (Cytiva, RPN303B) and UV-crosslinked (254 nm for 5 minutes). Then, the membrane was stained with 0.02% methylene blue in 0.3 M sodium acetate at pH 5.2. After imaging, the membrane was blocked with 5% BSA for 1 hour and incubated with anti-m^7^G antibody (MBL International, RN017M) overnight at 4°C. The next day, the membrane was incubated with HRP-conjugated anti-mouse secondary antibody for 1 hour. The m^7^G signals were developed using Amersham ECL Prime Western Blotting Detection Reagent (Cytiva, RPN2232).

### RIP-qRT-PCR.

Cell pellets were lysed in RIP lysis buffer on ice for 5 minutes, then incubated overnight at 4°C with rabbit IgG (Cell Signaling Technology, 2729) or anti-METTL1 antibody (Proteintech,14994-1-AP) conjugated to protein A/G magnetic beads to enrich RNA-protein complexes. After digestion with Proteinase K, RNA was purified by phenol-chloroform-isoamyl alcohol extraction and ethanol precipitation, then reverse-transcribed into cDNA. qRT-PCR was performed to determine the percentage of input of selected RNA candidates using the primers listed in Supplemental Table 5.

### Oxygen consumption.

Cells (1.50 × 10^5^) per well were plated on a Seahorse plate coated with Cell-Tak Cell and Tissue Adhesive (Corning Life Sciences) in XF 1640 (Agilent Technologies) containing 5.55 mM glucose, 4 mM glutamine, 1 mM sodium pyruvate, and oxygen consumption was recorded using a Seahorse XF96 Analyzer (Seahorse Biosciences). Each measurement was performed after a 3-minute mix and a 3-minute waiting period. Basal measurements were collected 4 times, followed by 4 measurements after addition of oligomycin (final concentration 2 μM), followed by 4 measurements after addition of FCCP (final concentration 3 μM), followed by 4 measurements after addition of rotenone plus antimycin A (final concentration 0.5 μM).

### Polysome profiling.

Polysome profiling was performed following previously described protocols with minor modifications(34). Cells were treated with 100 μg/mL cycloheximide (MilliporeSigma) at 37°C for 7 minutes before collection. Approximately 6 × 10^7^ cells per replicate were lysed in polysome lysis buffer (20 mmol/L HEPES, pH 7.6, 100 mmol/L KCl, 5 mmol/L MgCl_2_, 100 μg/mL cycloheximide, and 1% vol/vol Triton X-100), supplemented with 1:100 protease inhibitor (Thermo Fisher Scientific) and 40 U/mL SUPERase In RNase inhibitor (Thermo Fisher Scientific). Lysates were rotated at 4°C for 15 minutes, then centrifuged at 15,000*g* for 15 minutes at 4°C. The supernatant was collected and incubated with TURBO DNase (Thermo Fisher Scientific, AM2239) at room temperature for 15 minutes. Then, lysates were carefully loaded onto a 10% to 50% (w/v) sucrose gradient prepared in lysis buffer without Triton X-100 and centrifuged at 41,000 rpm at 4°C for 4 hours. Gradients were fractionated using a Piston Gradient Fractionator (Biocomp Instruments), monitored with an Econo UV detector (Bio-Rad), and collected with a fraction collector (Gilson). RNA was purified from fractions using QIAzol Lysis Reagent and analyzed with RT-qRT-PCR to quantify mRNA distribution across individual or pooled fractions. For RNA-seq, 10% of the lysate was reserved as input for total mRNA isolation. mRNA was fragmented by heating at 94°C for 25 minutes in 1× PNK (T4 polynucleotide kinase) buffer (New England Biolabs [NEB]). RNA fragments were end-repaired using T4 PNK (NEB, M0201S), purified using the RNA Clean and Concentrator kit (Zymo Research), and libraries were prepared using the NEBNext Ultra II Directional RNA Library Prep Kit for Illumina (NEB, E7760L). Libraries were sequenced on an Illumina NovaSeq X platform.

### Codon usage analysis.

Codon usage was analyzed using principal CDSs selected from GENCODE version 49 transcriptome annotations. Principal isoforms were identified by filtering transcripts against APPRIS rankings and retaining the highest-scoring isoform per gene. For each gene, per-codon raw counts were obtained using the coRdon package (version 1.16.0) and normalized to counts per 1,000 codons. Genes were classified as TE-down, TE-up, or NS (non-significant) according to the concordant changes in translation efficiency observed in polysome profiling. For each codon, log_2_ FCs were then calculated as log_2_(down/up) and log_2_(down/all), representing enrichment in translationally downregulated genes relative to TE-up genes and the genome-wide average, respectively. Valine codons (GTT, GTC, GTA, GTG) were specifically examined to assess m^7^G-codon enrichment. For individual codon comparisons across TE groups, per-gene log_2_-transformed frequencies were compared between TE-down and all other genes using the Wilcoxon rank-sum test. Combined m^7^G-modified valine codon usage was defined as the sum of the per-gene frequencies for GTT, GTA, and GTG. Results from the codon usage analysis are provided in Supplemental Table 4.

### Statistics.

All statistical analyses were conducted using SPSS Statistics (IBM Corporation) and GraphPad Prism (GraphPad Software). Results from experiments with at least 3 biological replicates are presented as the mean ± SD. For comparisons between 2 groups, either an independent or paired-samples 2-tailed *t* test was used, as appropriate. For comparisons among more than 2 groups, 1-way ANOVA (Bonferroni’s test or Dunnett’s test) was performed. Pearson correlation tests were performed. Kaplan-Meier survival curves were generated to evaluate DFS. Patients were stratified into high and low expression groups using the median expression value of METTL1 (or WDR4) as the cutoff. Survival differences between groups were assessed using the log-rank test. A *P* value of less than 0.05 was considered statistically significant.

### Study approval.

All participants provided informed written consent, and the IRB of Beckman Research Institute approved the study. Written informed consent was obtained from all patients prior to enrollment, and the IRB of Beckman Research Institute of City of Hope (Duarte, California, USA) approved the study protocol. The mouse study protocol was approved by the IACUC of the City of Hope.

### Data availability.

The data supporting the findings of this study are available within this article, in the supplemental material, and in the Supporting Data Values file. All data derived from the public database are available from these sites. All RNA-seq and polysome profiling datasets generated in this study have been deposited in the NCBI Gene Expression Omnibus (GEO) repository (GEO GSE329301). All other data reported in this article will be shared by the corresponding author upon request.

## Author contributions

JZ finished all experiments and wrote the manuscript. QZ performed bioinformatics analyses. DE provided the clinical samples. QP and RS provided a conceptual evaluation of this study. AG conceived the idea, designed the project, and edited the manuscript.

## Conflict of interest

The authors have declared that no conflict of interest exists.

## Funding support

This work is the result of NIH funding, in whole, and is subject to the NIH Public Access Policy. Through acceptance of this federal funding, the NIH has been given the right to make the work publicly available in PubMed Central.

- NIH grant R01CA181572 (to AG).
- NIH grant R01CA184792 (to AG).
- NIH grant U01CA187956 (to AG).
- NIH grant U01CA214254 (to AG).
- NIH grant R01CA271443 (to AG).
- NIH grant U01CA296639 (to AG).

## Supplementary Material

Supplemental data

Unedited blot and gel images

Supplemental tables 2-4

Supporting data values

## Figures and Tables

**Figure 1:**
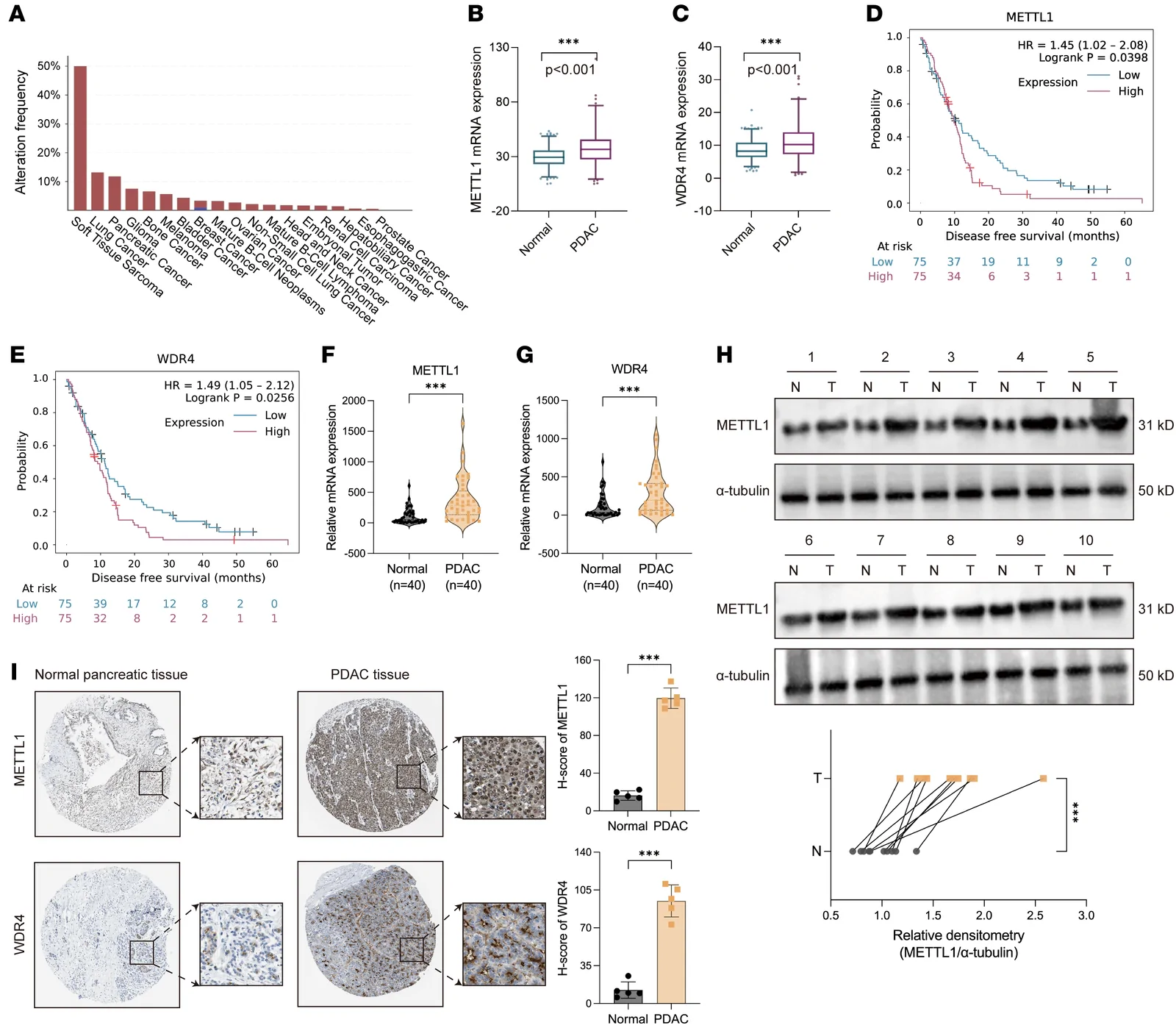
METTL1 is elevated in PDAC and correlates with a poor prognosis. (**A**) Alteration of METTL1 in human cancers from TCGA (CBioPortal). (**B** and **C**) Relative mRNA expression of *METTL1* (**B**) and *WDR4* (**C**) in pancreatic cancer and normal pancreatic tissues from TCGA and the GTEx databases. ***P < 0.001, by 2-tailed, unpaired *t* test. (**D**) Kaplan-Meier disease-free survival (DFS) analysis of PDAC patients with low or high METTL1 expression from TCGA database. Patients were divided into high and low expression groups using the median expression value as the cutoff. *P* = 0.0398, by log-rank (Mantel-Cox) test. (**E**) Kaplan-Meier DFS analysis of PDAC patients with low or high expression of WDR4 from TCGA database. Patients were divided into high and low expression groups using the median expression value as the cutoff. *P* = 0.0256, by log-rank (Mantel-Cox) test. (**F** and **G**) Relative mRNA expression levels of *METTL1* (**F**) and *WDR4* (**G**) were evaluated by qRT-PCR in a cohort of 40 paired PDAC and AN tissue specimens. ***P < 0.001, by 2-tailed, paired *t* test. (**H**) Western blot analysis of METTL1 protein expression in 10 paired PDAC and AN tissue samples and quantification of relative METTL1 protein levels normalized to α-tubulin. ***P < 0.001, by 2-tailed, paired *t* test. (**I**) Representative IHC images showing endogenous METTL1 and WDR4 expression in PDAC and normal pancreatic tissues from The Human Protein Atlas database, with corresponding H scores quantified and presented as bar graphs. Scale bar: 100 μm; original magnification, ×20; insets ×71. ***P < 0.001, by 2-tailed, unpaired *t* test. Data are representative of 3 independent experiments. All data are presented as the mean ± SD.

**Figure 2:**
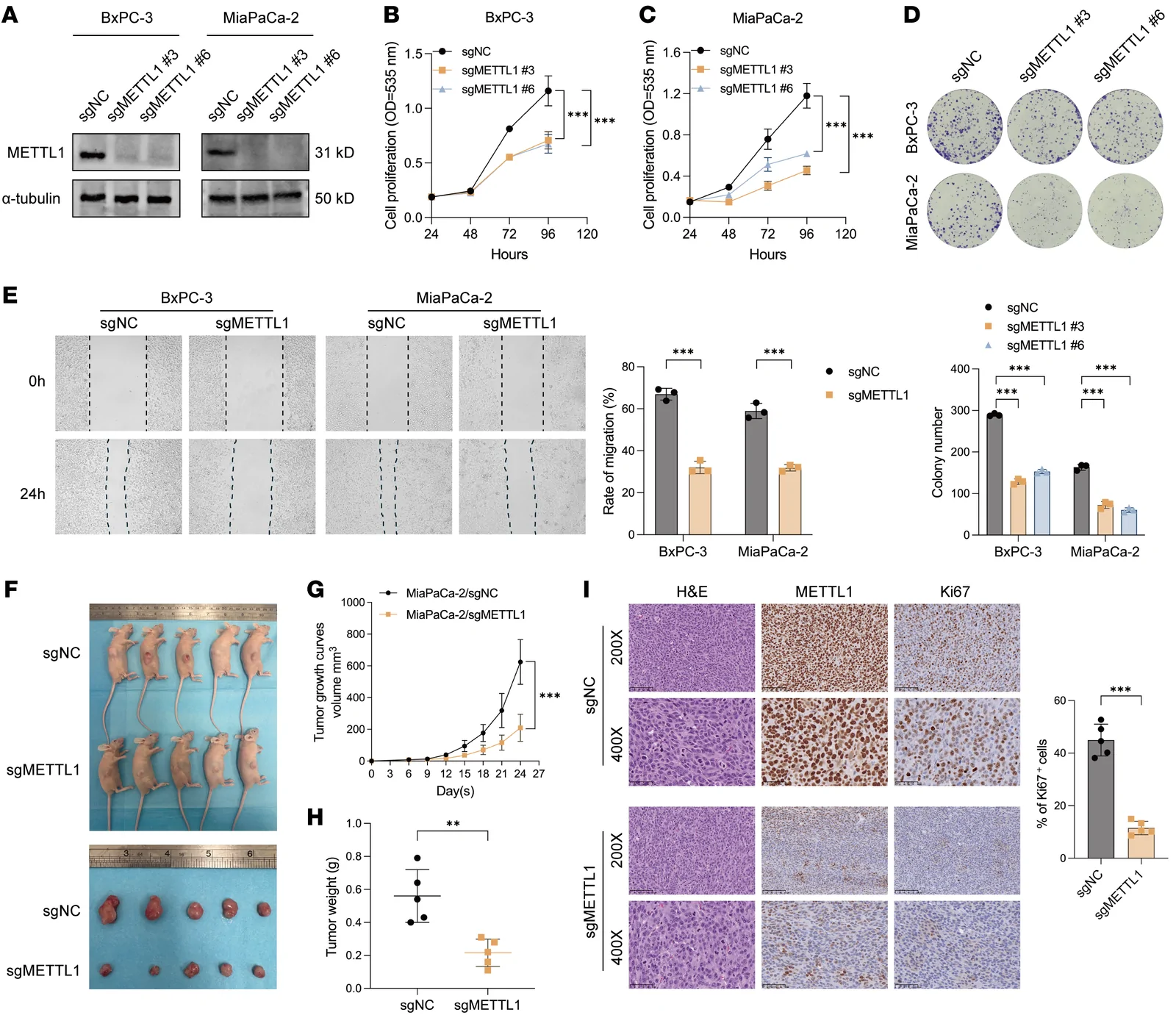
METTL1 promotes PDAC cell growth, migration, and tumorigenicity. (**A**) METTL1-KO efficacy in BxPC-3 and MiaPaCa-2 Cas9 single clones. (**B**–**D**) Detection of proliferative activity by MTT assay (**B** and **C**) and colony formation assay (**D**) in control and METTL1-KO groups from BxPC-3 and MiaPaCa-2 cells. ***P < 0.001, by 2-way ANOVA followed by Dunnett’s multiple-comparison test (**B** and **C**) and 1-way ANOVA followed by Dunnett’s multiple-comparison test (**D**). (**E**) Detection of migration activity by wound-healing assay of BxPC-3 and MiaPaCa-2 cells at 0 hours and 24 hours in the control and METTL1-KO groups. ***P < 0.001, by 2-tailed, unpaired *t* test. (**F**) Images of mice and s.c. xenograft tumors derived from MiaPaCa-2 control and METTL1-KO cells (*n* = 5 mice per group). (**G** and **H**) Tumor growth curves (**G**) and tumor weights (**H**) for nude mice injected with MiaPaCa-2 control or METTL1-KO cells. **P < 0.01 and ***P < 0.001, by 2-way, repeated-measures ANOVA (**G**) and 2-tailed, unpaired *t* test (**H**). (**I**) Representative IHC images of H&E, METTL1, and Ki67 staining of dissected xenograft tumor tissues and quantification of the percentage of Ki67^+^ cells (*n* = 5 tumors per group). Original magnification, ×200 and ×400. Scale bars: 100 μm and 50 μm. ***P < 0.001, by 2-tailed, unpaired *t* test. Data are representative of 3 independent experiments. All data are presented as the mean ± SD.

**Figure 3:**
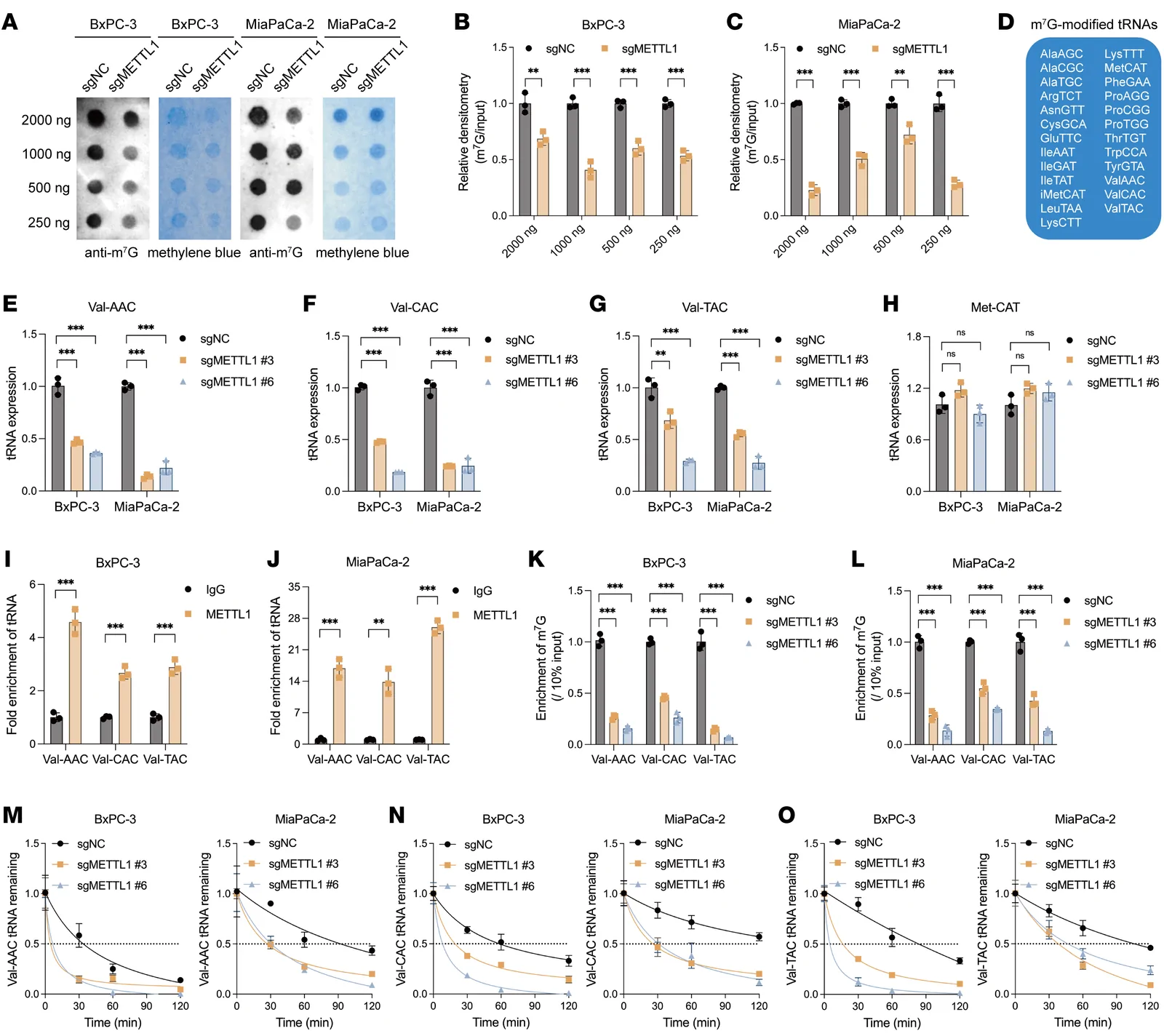
METTL1 depletion reduces the levels of m^7^G-modified valine tRNAs by destabilizing them. (**A**) Dot blot analysis of global m^7^G RNA modification levels in control and METTL1-KO groups from BxPC-3 and MiaPaCa-2 cells. (**B** and **C**) Densitometric quantification of m^7^G dot blot signals normalized to input RNA at each concentration in BxPC-3 (**B**) and MiaPaCa-2 (**C**) cells. **P < 0.01 and ***P < 0.001, by 2-tailed, unpaired *t* test. (**D**) Subset of m^7^G-modified tRNAs identified in human cells. (**E**–**H**) Relative expression of candidate tRNAs, Val-AAC (**E**), Val-CAC (**F**), Val-TAC (**G**), and Met-CAT (**H**) in control and METTL1-KO groups from BxPC-3 and MiaPaCa-2 cells by qRT-PCR. **P < 0.01 and ***P < 0.001, by 1-way ANOVA followed by Dunnett’s multiple-comparison test. (**I** and **J**) RIP assays with anti-METTL1 antibody or IgG control followed by qRT-PCR to detect valine tRNA enrichment in BxPC-3 (**I**) and MiaPaCa-2 (**J**) cells. **P < 0.01 and ***P < 0.001, by 2-tailed, unpaired *t* test. (**K** and **L**) The m^7^G modification of valine tRNAs was detected in the control and METTL1-KO groups from BxPC-3 (**K**) and MiaPaCa-2 (**L**) cells through MeRIP-qRT-PCR using an anti-m^7^G antibody. ***P < 0.001, by 1-way ANOVA, followed by Dunnett’s multiple-comparison test. (**M**–**O**) Relative expression of candidate tRNAs, Val-AAC (**M**), Val-CAC (**N**), and Val-TAC (**O**) by qRT-PCR in BxPC-3 and MiaPaCa-2 control and METTL1-KO cells treated with 5 μg/mL actinomycin D for the indicated durations. Data are representative of 3 independent experiments. All data are presented as the mean ± SD.

**Figure 4:**
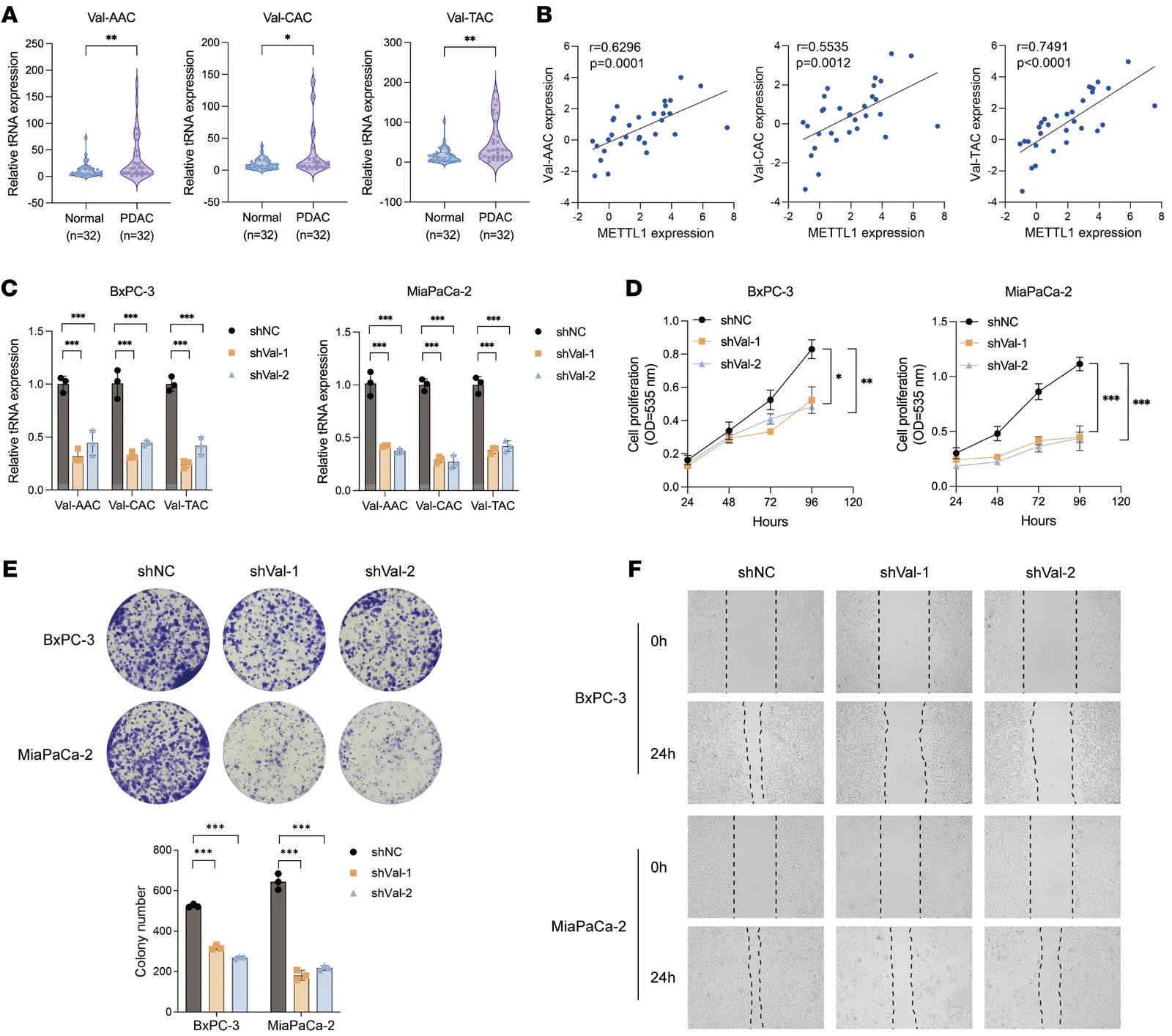
Valine tRNAs are upregulated in PDAC and are necessary for cancer cell growth and migration. (**A**) The relative expression of valine tRNAs was evaluated by qRT-PCR in a cohort of 32 paired PDAC and AN tissue specimens. *P < 0.05 and **P < 0.01, by 2-tailed, paired *t* test. (**B**) Pearson correlation analysis of METTL1 and valine tRNA expression levels. (**C**) The relative expression of candidate tRNAs was determined by qRT-PCR in control and valine tRNA–KD groups of BxPC-3 and MiaPaCa-2 cells. ***P < 0.001, by 1-way ANOVA followed by Dunnett’s multiple-comparison test. (**D** and **E**) Detection of proliferative activity by MTT assay (**D**) and colony formation assay (**E**) in control and valine tRNA–KD groups of BxPC-3 and MiaPaCa-2 cells. *P < 0.05, **P < 0.01, and ***P < 0.001, by 2-way ANOVA (**D**) and 1-way ANOVA (**E**) followed by Dunnett’s multiple-comparison test. (**F**) Detection of migration activity by wound-healing assay in control and valine tRNA–KD group of BxPC-3 and MiaPaCa-2 cells at 0 and 24 hours. Data are representative of 3 independent experiments. Original magnification, ×1 (**E**), ×6.3 (**F**). All data are presented as the mean ± SD.

**Figure 5:**
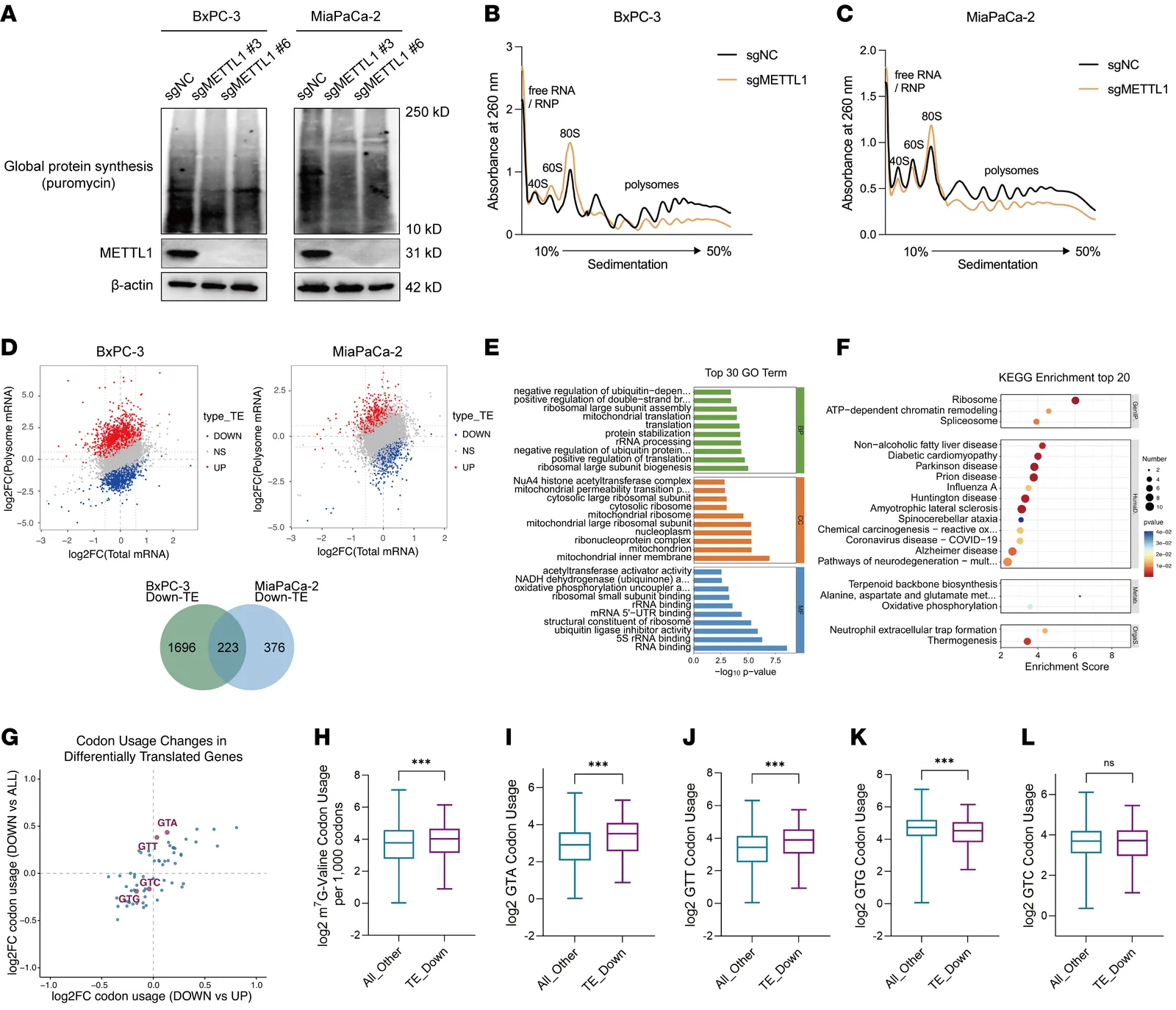
METTL1 depletion leads to valine codon–biased changes in mRNA translation. (**A**) Western blotting analysis of global protein synthesis in the control and METTL1-KO groups from BxPC-3 and MiaPaCa-2 cells. (**B** and **C**) Ribosome fractionation by sucrose density gradient centrifugation in the control and METTL1-KO groups from BxPC-3 (**B**) and MiaPaCa-2 (**C**) cells. (**D**) Scatterplot of TE in METTL1-KO versus control cells (Up). TE was calculated by dividing the polysome-binding signals by the input RNA-seq signals. Venn diagram of overlapping genes with downregulated TE (Down-TE) in BxPC-3 and MiaPaCa-2 cells (down). (**E** and **F**) GO (**E**) and KEGG pathway (**F**) enrichment analyses of the overlapping genes with downregulated TE in BxPC-3 and MiaPaCa-2 cells. (**G**) Scatterplot of codon use changes in differentially translated genes (down vs. all other and down vs. up) in METTL1-KO versus control cells. Valine codons are highlighted in magenta. (**H**) Comparison of m^7^G-valine codon usage between TE-down genes versus all other genes. ***P < 0.001, by Wilcoxon rank-sum test. (**I**–**L**) Comparison of individual valine codon usage between TE-down genes versus all other genes. (**I**) GTA. (**J**) GTT. (**K**) GTG. (**L**) GTC. ***P < 0.001, by Wilcoxon rank-sum test. Data are representative of 3 independent experiments. All data are presented as the mean ± SD.

**Figure 6:**
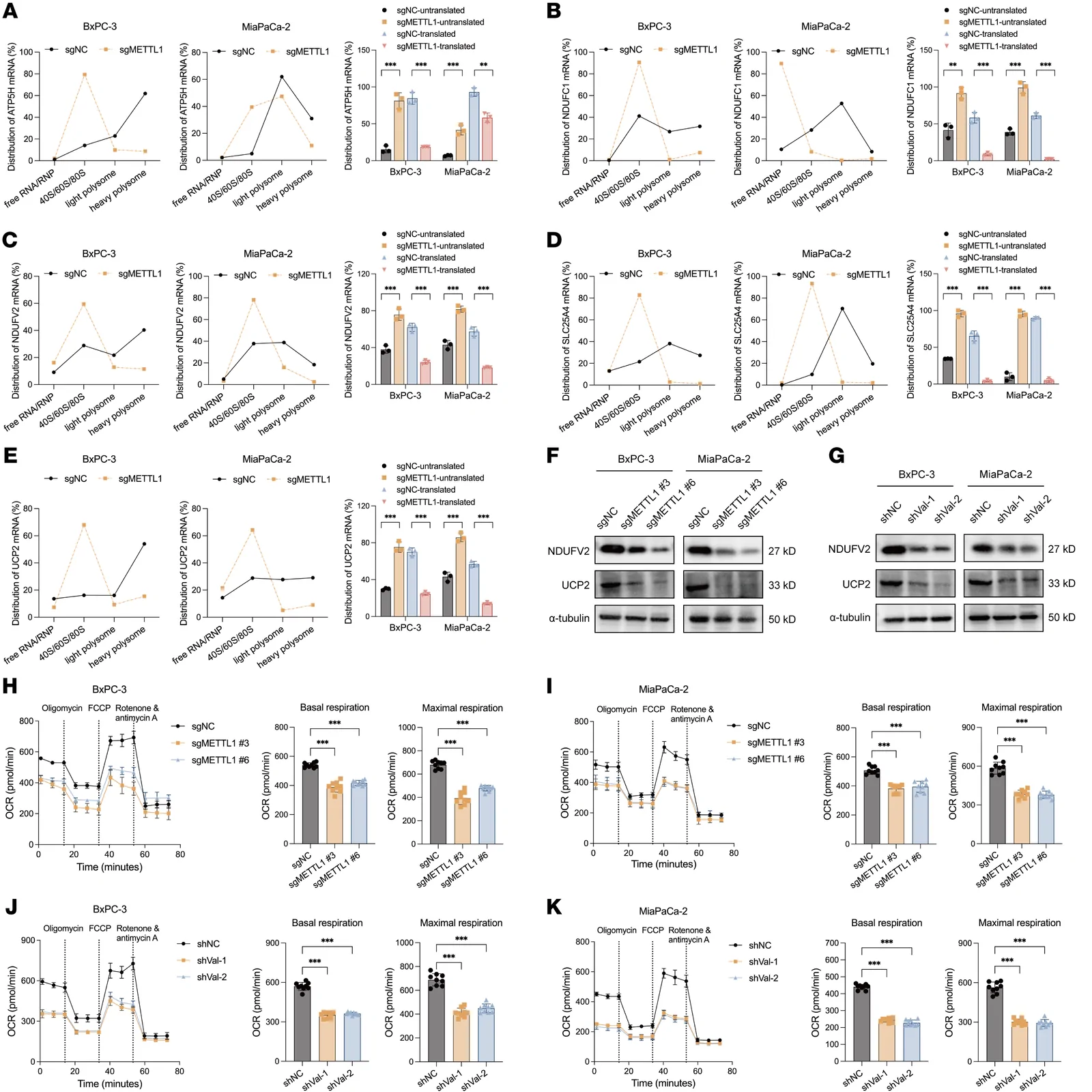
METTL1/valine tRNA depletion impairs mitochondrial bioenergetics in PDAC cells. (**A**–**E**) Distribution of *ATP5H* (**A**), *NDUFC1* (**B**), *NDUFV2* (**C**), *SLC25A4* (**D**), and *UCP2* (**E**) mRNAs across ribosome fractions (free RNA/RNP, 40S/60S/80S, light polysome, heavy polysome) from sgNC and sgMETTL1 BxPC-3 and MiaPaCa-2 cells, quantified by qRT-PCR. Bar graphs show the proportion of untranslated and translated fractions. **P < 0.01 and ***P < 0.001, by 2-tailed, unpaired *t* test. (**F**) Relative protein levels of NDUFV2 and UCP2 in PDAC control and METTL1-KO cells by Western blotting. (**G**) Relative protein levels of NDUFV2 and UCP2 in PDAC control and valine tRNA–KD cells by Western blotting. (**H** and **I**) Oxygen consumption rate in control and METTL1-KO groups from BxPC-3 (**H**) and MiaPaCa-2 (**I**) cells by Seahorse Mito Stress testing. ***P < 0.001, by 1-way ANOVA followed by Dunnett’s multiple-comparison test. (**J** and **K**) Oxygen consumption rate in control and valine tRNA–KD group from BxPC-3 (**J**) and MiaPaCa-2 (**K**) cells by Seahorse Mito Stress testing. ***P < 0.001, by1-way ANOVA followed by Dunnett’s multiple-comparison test. Data are representative of 3 independent experiments. All data are presented as the mean ± SD.

**Figure 7:**
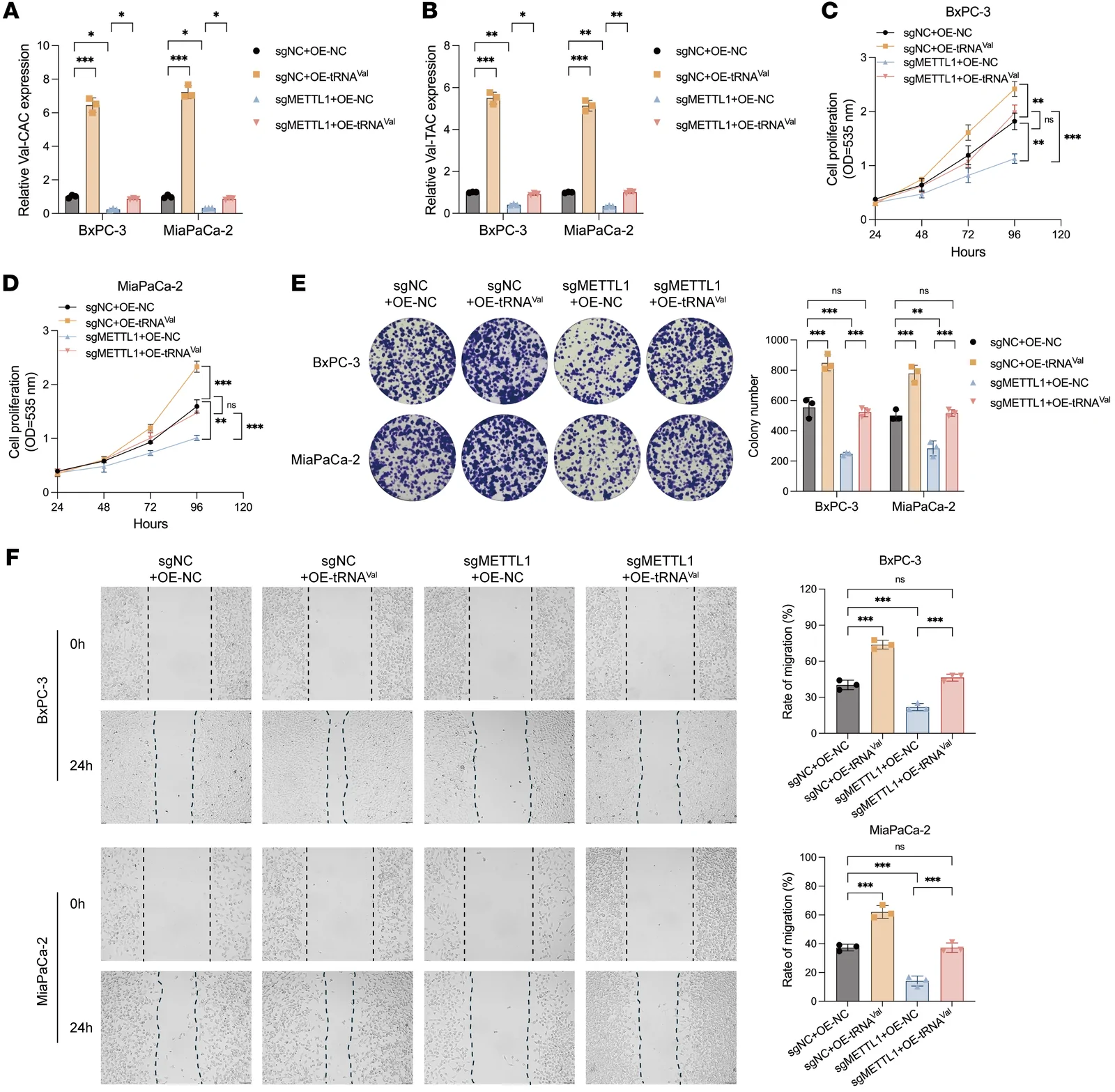
Overexpression of valine tRNAs recapitulates METTL1-driven oncogenic changes. (**A** and **B**) Relative expression of Val-CAC (**A**) and Val-TAC (**B**) was evaluated by qRT-PCR in PDAC control and METTL1-KO cells cotransfected with empty vector or valine tRNA–overexpressing plasmids. *P < 0.05, **P < 0.01, and ***P < 0.001, by1-way ANOVA followed by Tukey’s multiple-comparison test. (**C**–**E**) Detection of proliferative activity by MTT assay (**C** and **D**) and colony formation assay (**E**) in PDAC control and METTL1-KO cells cotransfected with empty vector or valine tRNA–overexpressing plasmids. **P < 0.01 and ***P < 0.001, by 2-way ANOVA (**C** and **D**) and 1-way ANOVA (**E**) followed by Tukey’s multiple-comparison test. (**F**) Detection of migration activity by wound-healing assay in PDAC control and METTL1-KO cells cotransfected with empty vector or valine tRNA–overexpressing plasmids at 0 and 24 hours. Original magnification, ×1 (**E**), ×6.3 (**F**). ***P < 0.001, by 1-way ANOVA followed by Tukey’s multiple-comparison test. Data are representative of 3 independent experiments. All data are presented as the mean ± SD.

## Version 1 08/11/2026

In-Press Preview

## Version 2 10/01/2026

Electronic publication
