# Supplementary material for: METTL1-mediated m7G modification of valine tRNAs drives metabolic adaptation in pancreatic ductal adenocarcinoma cells
Source: J Clin Invest. 2026 Aug 6;136(19):e204312. doi: 10.1172/JCI204312 (PMC13626843; doi:10.1172/JCI204312)
Supplement: Supplementary file 1 [file jci-136-204312-s293.pdf]

Supplemental material

METTL1-mediated m7G modification of valine tRNAs drives metabolic adaptation in pancreatic ductal adenocarcinoma cells

Jiabei Zhu[1,2], Qi Zhang[2], Douglas Evans[3], Rui Su[4], Qiuhui Pan[2,*], Ajay Goel[1,5,*]

1. Department of Molecular Diagnostics and Experimental Therapeutics, Beckman Research Institute of City of Hope, Biomedical Research Center, Monrovia, CA, USA
2. Department of Clinical Laboratory, Shanghai Children's Medical Center, Shanghai Jiao Tong University School of Medicine, Shanghai, China.
3. Department of Surgery, Medical College of Wisconsin, Milwaukee, WI, USA.
4. Department of Systems Biology, Beckman Research Institute of City of Hope, Monrovia, CA USA
5. City of Hope Comprehensive Cancer Center, Duarte, CA, USA

Supplemental figures

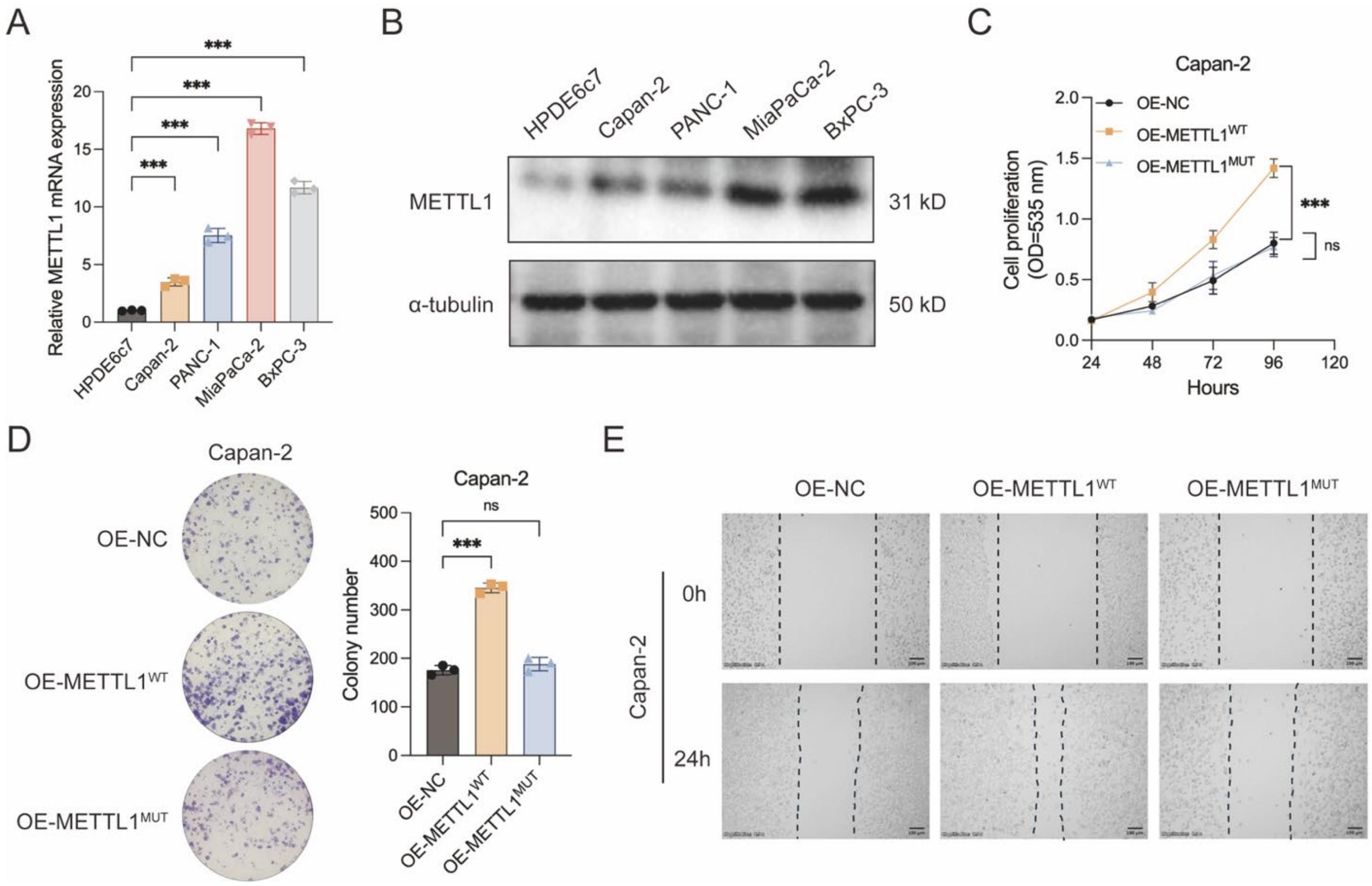

Figure S1. METTL1 expression in PDAC cell lines and gain-of-function analysis in Capan-2 cells. (**A**) Relative METTL1 mRNA expression assessed by qRT-PCR in human pancreatic duct epithelial HPDE6c7 cells and PDAC cell lines (Capan-2, PANC-1, MiaPaCa-2, BxPC-3). One-way ANOVA followed by Dunnett's multiple comparisons test. (**B**) Western blotting analysis of METTL1 protein expression in HPDE6c7 and PDAC cell lines. **(C-D)** Detection of proliferative activity by MTT assay (C) and colony formation assay (D) in in Capan-2 cells overexpressing empty vector (OE-NC), wild-type METTL1 (OE-METTL1^{WT}), or catalytically inactive METTL1 mutant (OE-METTL1^{MUT}). C, Two-way ANOVA followed by Dunnett's multiple comparisons test. D, One-way ANOVA followed by Dunnett's multiple comparisons test. (**E**) Wound healing assay evaluating cell migration in Capan-2 OE-NC, OE-METTL1^{WT}, and OE-METTL1^{MUT} cells at 0 h and 24 h. Data are representative of three independent experiments. *$p < 0.05$, **$p < 0.01$, ***$p < 0.001$.

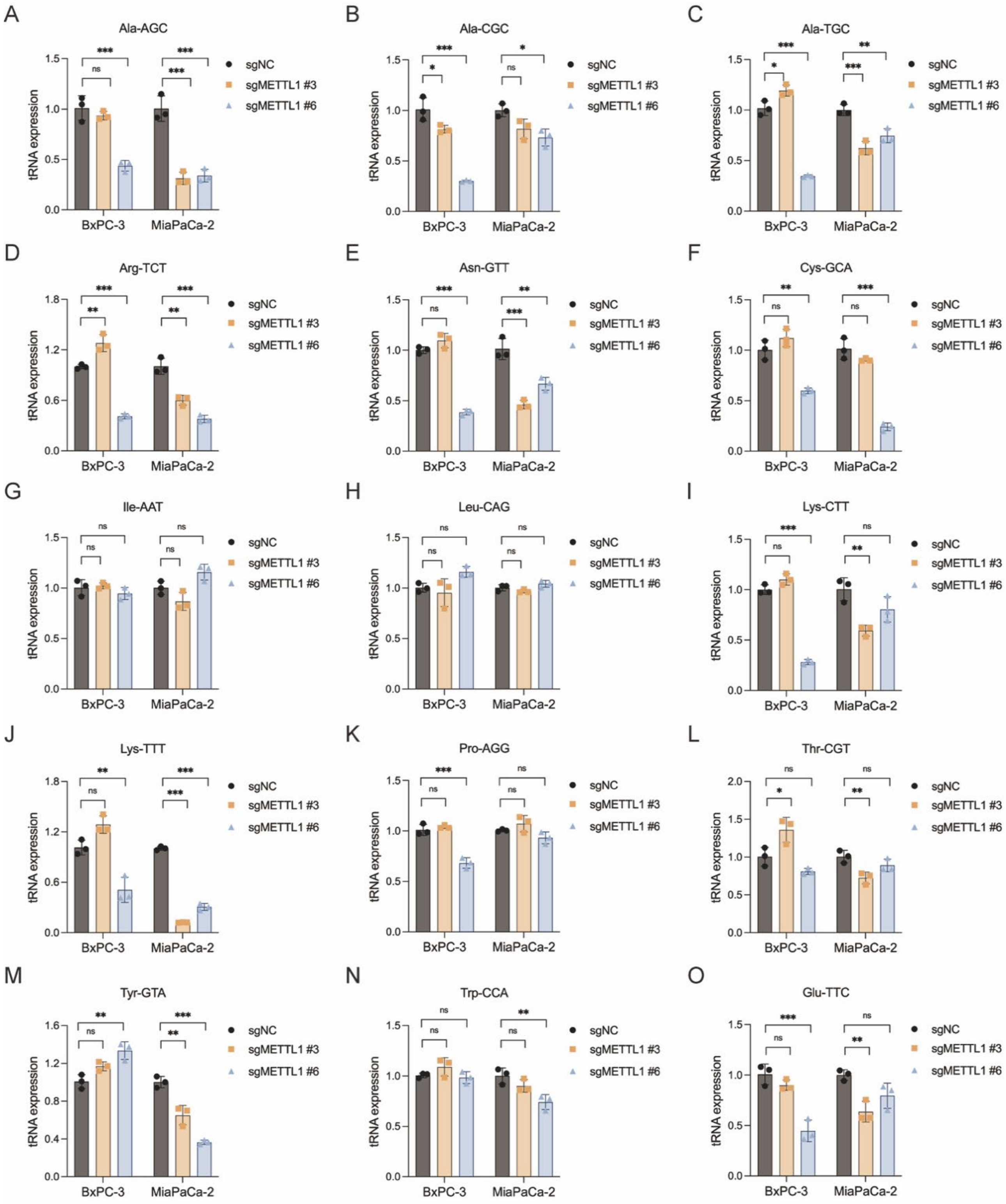

Figure S2. The effect of METTL1 depletion on the expression of non-valine tRNA species in PDAC cells. (**A-O**) Relative expression of Ala-AGC (A), Ala-CGC (B), Ala-TGC (C), Arg-TCT (D), Asn-GTT (E), Cys-GCA (F), Ile-AAT (G), Leu-CAG (H), Lys-CTT (I), Lys-TTT (J), Pro-AGG (K), Thr-CGT (L), Tyr-GTA (M), Trp-CCA (N), and Glu-TTC (O) tRNA species measured by qRT-PCR in sgNC and sgMETTL1 BxPC-3 and MiaPaCa-2 cells. Data are representative of three independent experiments. One-way ANOVA followed by Dunnett's multiple comparisons test. *$p < 0.05$, **$p < 0.01$, ***$p < 0.001$.

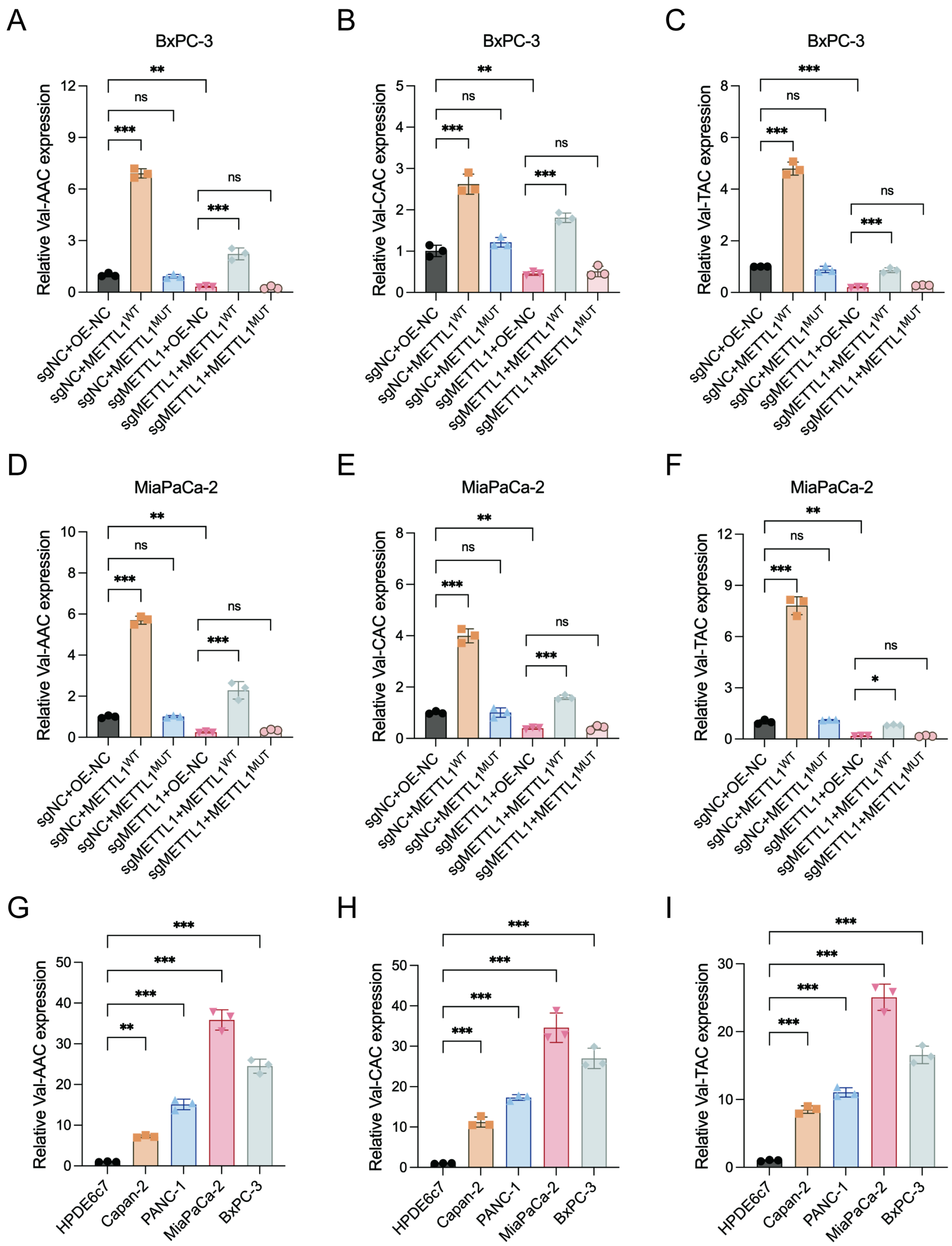

Figure S3. **METTL1 methyltransferase activity is required for valine tRNA regulation.** (**A-C**) Relative expression of Val-AAC (A), Val-CAC (B), and Val-TAC (C) by qRT-PCR in BxPC-3 sgNC and sgMETTL1 cells transfected with OE-NC, OE-METTL1^{WT}, or OE-METTL1^{MUT}. One-way ANOVA followed by Tukey's multiple comparisons test. (**D-F**) Relative expression of Val-AAC (D), Val-CAC (E), and Val-TAC (F) by qRT-PCR in MiaPaCa-2 sgNC and sgMETTL1 cells transfected with OE-NC, OE-METTL1^{WT}, or OE-METTL1^{MUT}. One-way ANOVA followed by Tukey's multiple comparisons test. (**G-I**) Relative expression of Val-AAC (G), Val-CAC (H), and Val-TAC (I) assessed by qRT-PCR in HPDE6c7 and PDAC cell lines. One-way ANOVA followed by Dunnett's multiple comparisons test. Data are representative of three independent experiments. *$p < 0.05$, **$p < 0.01$, ***$p < 0.001$.

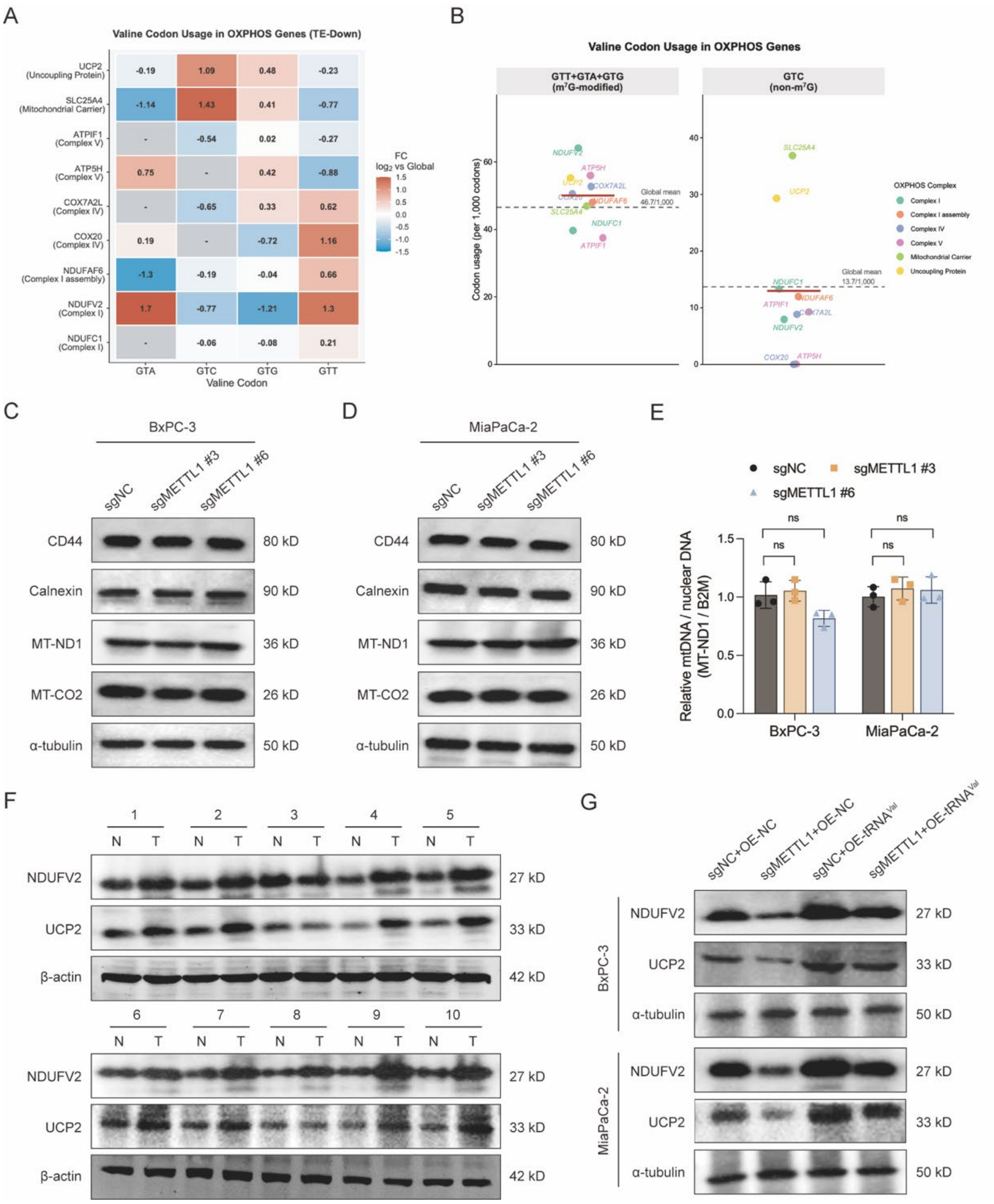

Figure S4. Codon-specific translational effects of METTL1 loss on OXPHOS genes. (**A**) Valine codon usage (log2 fold change vs. genome-wide average) for the TE-down OXPHOS genes across the four valine codons (GTA, GTC, GTG, GTT). (**B**) Bubble plot of GTT+GTA+GTG (m^7G-modified) and GTC (non-m^7G) codon usage per 1,000 CDS codons for each TE-down OXPHOS gene. Dashed lines indicate the genome-wide average for each codon group. (**C-D**) Western blotting analysis of CD44, Calnexin, MT-ND1, and MT-CO2 protein levels in sgNC and sgMETTL1 BxPC-3 (C) and MiaPaCa-2 (D) cells. (**E**) Relative mtDNA content in sgNC and sgMETTL1 BxPC-3 and MiaPaCa-2 cells, determined by qPCR analysis of the MT-ND1/B2M copy number ratio. One-way ANOVA followed by Dunnett's multiple comparisons test. (**F**) Western blotting analysis of NDUFV2 and UCP2 protein levels in 10 paired PDAC and AN tissues. (**G**) Western blotting analysis of NDUFV2 and UCP2 protein levels in sgNC and sgMETTL1 cells transfected with empty vector or valine tRNA overexpression plasmids. Data are representative of three independent experiments. *p < 0.05, **p < 0.01, ***p < 0.001.

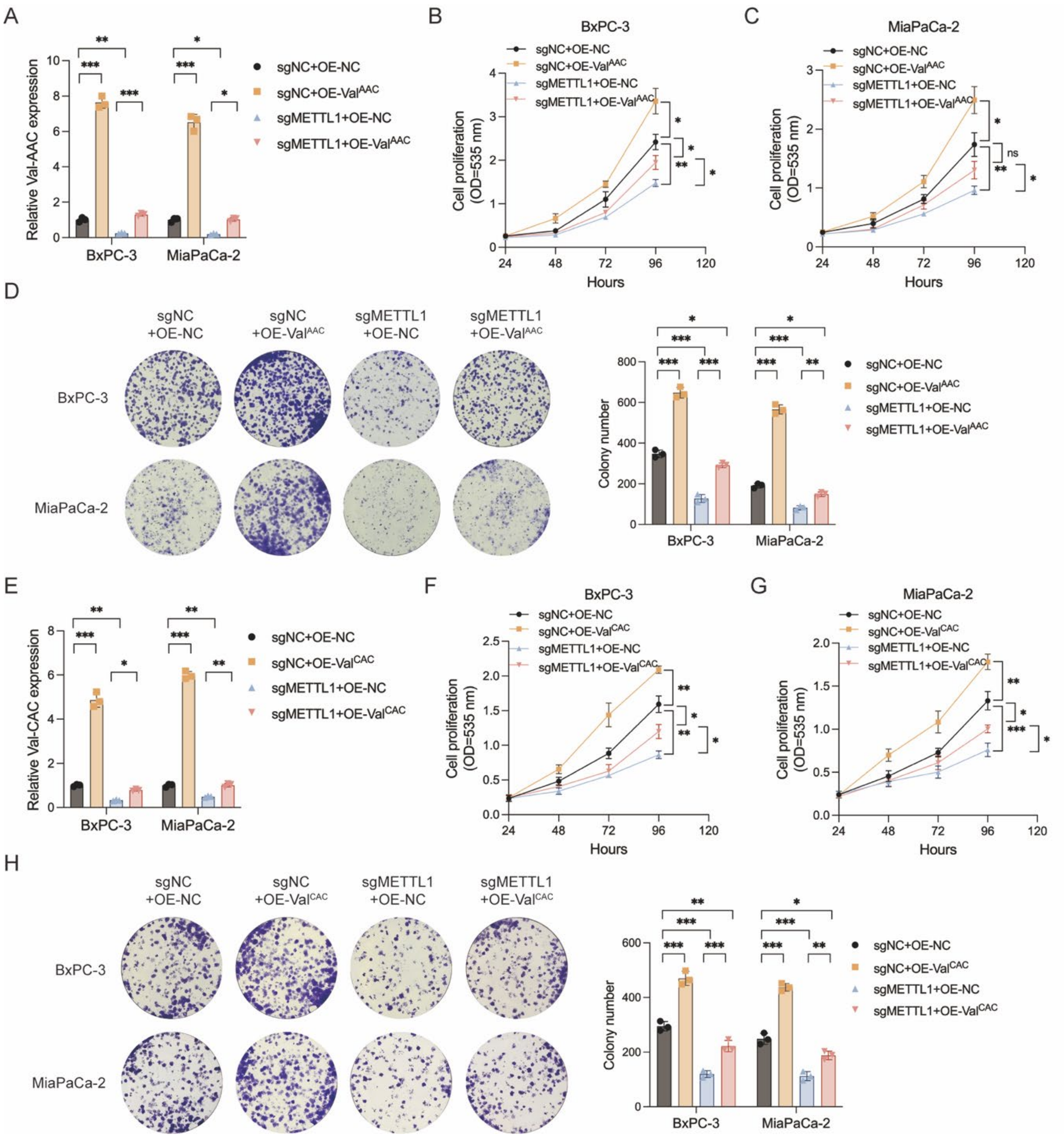

Figure S5. Overexpression of an individual valine tRNA partially rescues METTL1-KO phenotypes. (**A**) Relative Val-AAC expression assessed by qRT-PCR in sgNC and sg-METTL1 BxPC-3 and MiaPaCa-2 cells transfected with empty vector (OE-NC) or Val-AAC overexpression plasmid (OE-Val^{AAC}). One-way ANOVA followed by Tukey's multiple comparisons test. (**B-D**) Cell proliferation assessed by MTT assay (B-C) and colony formation assay (D) in sgNC and sg-METTL1 BxPC-3 and MiaPaCa-2 cells transfected with OE-NC or OE-Val^{AAC}. B-C, Two-way ANOVA followed by Tukey's multiple comparisons test. D, One-way ANOVA followed by Tukey's multiple comparisons test. (**E**) Relative Val-CAC expression assessed by qRT-PCR in sgNC and sg-METTL1 BxPC-3 and MiaPaCa-2 cells transfected with OE-NC or Val-CAC overexpression plasmid (OE-Val^{CAC}). One-way ANOVA followed by Tukey's multiple comparisons test. (**F-H**) Cell proliferation assessed by MTT assay (F-G) and colony formation assay (H) in sgNC and sg-METTL1 BxPC-3 and MiaPaCa-2 cells transfected with OE-NC or OE-Val^{CAC}. F-G, Two-way ANOVA followed by Tukey's multiple comparisons test. H, One-way ANOVA followed by Tukey's multiple comparisons test. Data are representative of three independent experiments. *$p < 0.05$, **$p < 0.01$, ***$p < 0.001$.

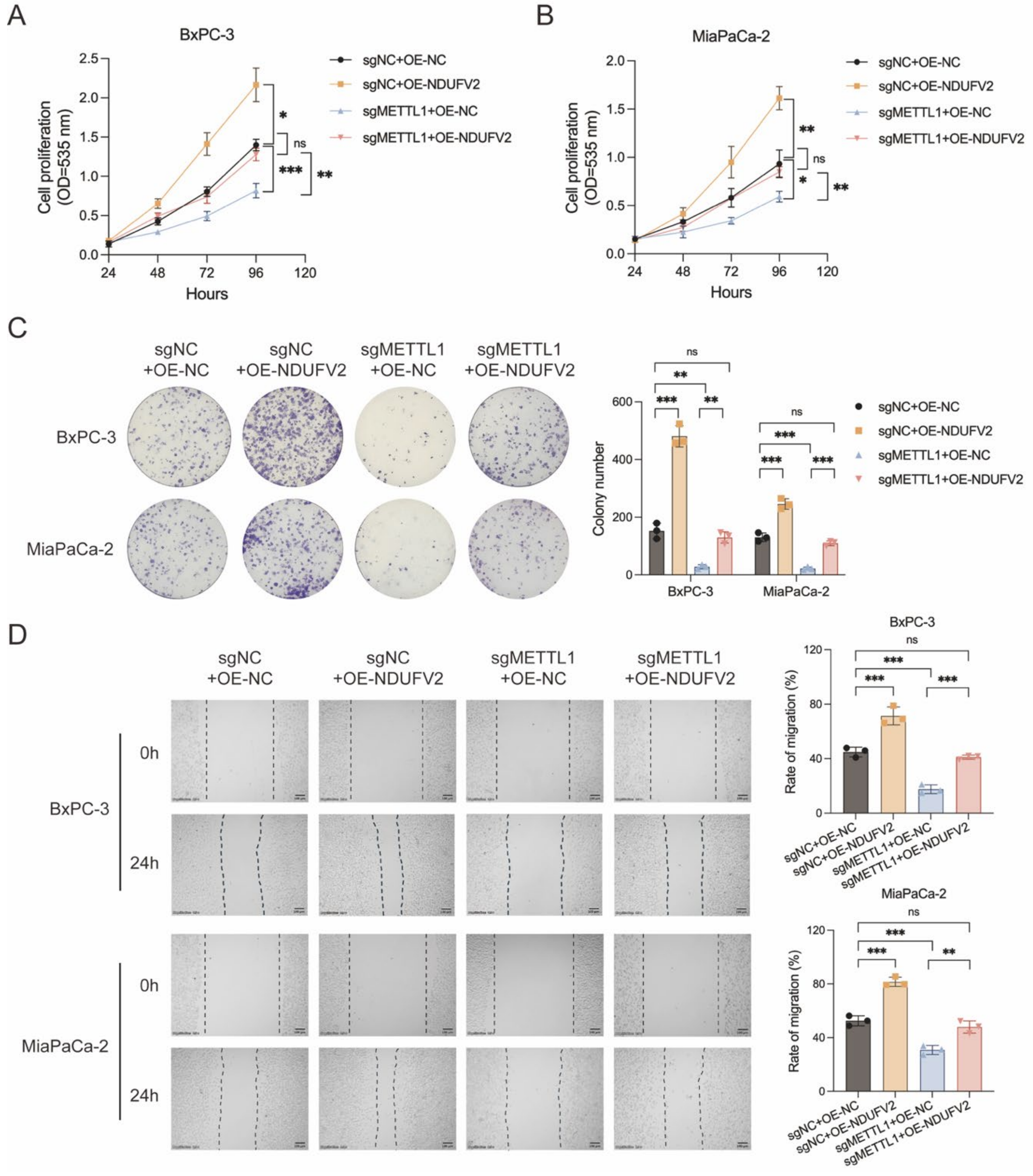

Figure S6. NDUFV2 overexpression rescues proliferation and migration defects in METTL1-KO cells. (**A-C**) Cell proliferation assessed by MTT assay (A-B) and colony formation assay (C) in sgNC and sgMETTL1 BxPC-3 and MiaPaCa-2 cells transfected with empty vector (OE-NC) or NDUFV2 overexpression plasmid (OE-NDUFV2). A-B, Two-way ANOVA followed by Tukey's multiple comparisons test. C, One-way ANOVA followed by Tukey's multiple comparisons test. (**D**) Wound healing assay evaluating cell migration in sgNC and sgMETTL1 BxPC-3 and MiaPaCa-2 cells transfected with OE-NC or OE-NDUFV2 at 0 h and 24 h. Rate of migration (%) is shown in bar graphs. One-way ANOVA followed by Tukey's multiple comparisons test. Data are representative of three independent experiments. *$p < 0.05$, **$p < 0.01$, ***$p < 0.001$.

Supplemental tables

Table S1. Patient characteristics of the clinical cohort

Characteristics	Total (n =40)
Age, mean (±SD) (years)	65
Sex	
Male	24
Female	16
Tumor location	
Head	21
Body	12
Tail	7
Tumor size, mean (±SD) (mm)	34
T stage	
1	2
2	11
3	26
4	1
Lymph node metastasis	
Negative	30
Positive	10
Distant metastasis	
Negative	18
Positive	22
Stage	
I	1
II	11
III	6
IV	22
CEA (ng/mL)	
> 5.0	17
≤ 5.0	23
CA19-9 (U/mL)	
> 37.0	35
≤ 37.0	5
Death	
No	13
Yes	27

Table S5. List of primers for RT-qPCR used in this study.

Gene	Forward (5'-3')	Reverse (5'-3')
METTL1	GGCAACGTGCTCACTCCAA	CACAGCCTATGTCTGCAAACT
WDR4	GTTCGACAACGTGACCTCCT	TCCCCCGGTCTCATCTTCTT
Val-AAC	GGTTATCACGTTCGCCTAAC	AACCGGGGACCTTTCGCGTG
Val-CAC	GGTTATCACGTTCGCCTCAC	AACCGGGGACCTTTCGCGTG
Val-TAC	GGTTATCACGTCTGCTTTAC	AACCCAGGACCTTCTGCGTG
Ala-AGC	AGTGGTAGAGCGCGTGCTTA	CCGGGGCCTCGTGCATGCT
Ala-CGC	TGGTAGAGCGCATGCTTCGC	AACCCGGGACCTCATACATG
Ala-TGC	TGGTAGAGCGCATGCTTTGC	AACCCGGGGCCTCATACATG
Arg-TCT	ATGGATAGCGCATTGGACTT	CCCACAACCTTTGAATTAGA
Asn-GTT	GGTTAGCGCGTTCGGCTGTT	AACCACCAACCTTTCGGTTA
Cys-GCA	GGTAGAGCATTTGACTGCAG	GAACCAGGGACCTCTTGATC
Ile-AAT	GGTTAGAGCGTGGTGCTAAT	AACCCGCGACCTTGGCGTTA
Leu-CAG	GTCTAAGGCGCTGCGTTCAG	CTCCAGGGGAGACTGCGACC
Lys-CTT	CGGTAGAGCATGAGACTCTT	AACCCATGACCCTGAGATTA
Met-CAT	AGGCAGCGCGTCAGTCTCAT	AACTCACGACCTTCAGATTA
Pro-AGG	GGGTATGATTCTCGCTTAGG	AACCCGGGACCTCTCGCACC
Thr-CGT	TGGTAAGGCGTCGGTCTCGT	AACCCGCGATCTTCGGTTTA
Tyr-GTA	TGGTAGAGCGGAGGACTGTA	AACCAGCGACCTAAGGATCT
Trp-CCA	CGGTAGCGCGTCTGACTCCA	AACACGCAACCTTCTGATCT
Glu-TTC	GGTTAGGATTCCTGGTTTTC	GAACCCGGGCCACCTGGGTG
SLC25A4	CCTCACTAACCTACCCGCAC	GAGTCGGACGCTCAATTCCA
UCP2	GGGATTGACTGTCCACGCTC	CCGATCCCCTTGGTTTTCCA
ATP5H	TAATGCGCTGAAGGTTCCCG	GGCCTTTGAGAGAGACACCC
NDUFC1	GCAACTGACTACAAGGAGATGC	GCTCAGTCTCTCCGAGTTGG
NDUFV2	TAGCCCAAAGGCAGAATGGG	TCGAAGCATGCAGGGTGTAG
MT-ND1	CCCTAAAACCCGCCACATCT	GAGCGATGGTGAGAGCTAAGGT

B2M	TGCTGTCTCCATGTTTGATGTATCT	TCTCTGCTCCCCACCTCTAAGT
28S rRNA	CAGGGGAATCCGACTGTTTA	ATGACGAGGCATTTGGCTAC
Actin	CACTCTTCCAGCCTTCCTTC	GTACAGGTCTTTGCGGATGT

Table S6. List of oligonucleotides used in this study.

Name	Sequence (5'-3')
scramble SgRNA F	CACCGTGCGAATACGCCCACGCGATGGG
scramble SgRNA R	AAACCCCATCGCGTGGGCGTATTCGCAC
SgMETTL1-3 F	CACCGCCCTGCTAGCAGAATATGCC
SgMETTL1-3 R	AAACGGCATATTCTGCTAGCAGGGC
SgMETTL1-6 F	CACCGAAGTGGAGTTTGCAGACATA
SgMETTL1-6 R	AAACTATGTCTGCAAACTCCACTTC
ShRNA-Val-1F	CCGGGTAGTGTAGTGGTTATCACGTCTCGAGACGTGATAACCACTACACTACTTTTTG
ShRNA-Val-1R	AATTCAAAAAGTAGTGTAGTGGTTATCACGTCTCGAGACGTGATAACCACTACACTAC
ShRNA-Val-2F	CCGGGTTTCCGTAGTGTAGTGGTTACTCGAGTAACCACTACACTACGGAAACTTTTTG
ShRNA-Val-2R	AATTCAAAAAGTTTCCGTAGTGTAGTGGTTACTCGAGTAACCACTACACTACGGAAAC
Val-AAC-clone-F	CAACGTGAATTCACCCAAAGGAAGGGCAACTC
Val-AAC-clone-R	CAACGTACCGGTTGTCTTTTGAAGGACCGGCA
Val-CAC-clone-F	CAACGTGAATTCAACTCCACGACGAGCATTCG
Val-CAC-clone-R	CAACGTACCGGTCGGACCTAGAGTTCCGGGAT
3×FLAG METTL1 XBaI F	TGCTCTAGAGCCACCATGGACTACAAAGACCATGACGGTGATTATAAAGATCATGACATCGACTACAAGGATGACGATGACAAGGCAGCCGAGACTCGGA
METTL1 NotI R	AAATATGCGGCCGCTCAGTGACCAGGCAGGCTGGTTTG
METTL1 EIR/AAA F_In fusion	CTGGCGGCCGCGGTGAAGGTCTCAGACTATGTACAAGACC
METTL1 EIR/AAA R_In fusion	CACCGCGGCCGCCAGACCCAGAATAAGTGTGTCTGGGAAC
