# Supplementary material for: METTL1-mediated m7G modification of valine tRNAs drives metabolic adaptation in pancreatic ductal adenocarcinoma cells
Source: J Clin Invest. 2026 Aug 6;136(19):e204312. doi: 10.1172/JCI204312 (PMC13626843; doi:10.1172/JCI204312)

Full unedited blot for Figure 1H

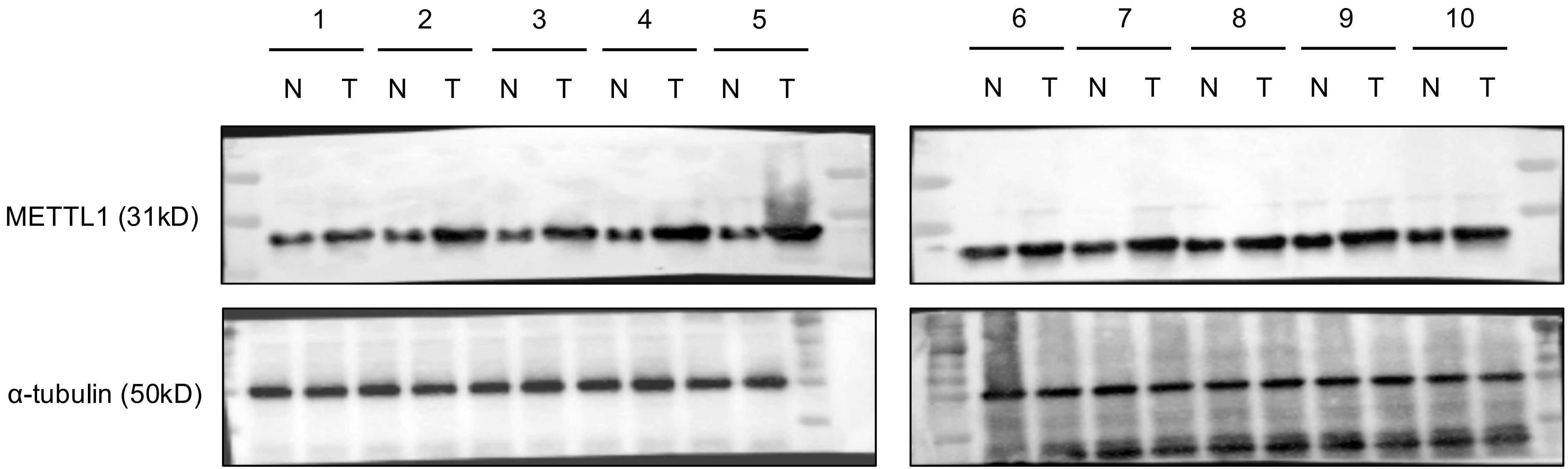

Full unedited blot for Figure 2A

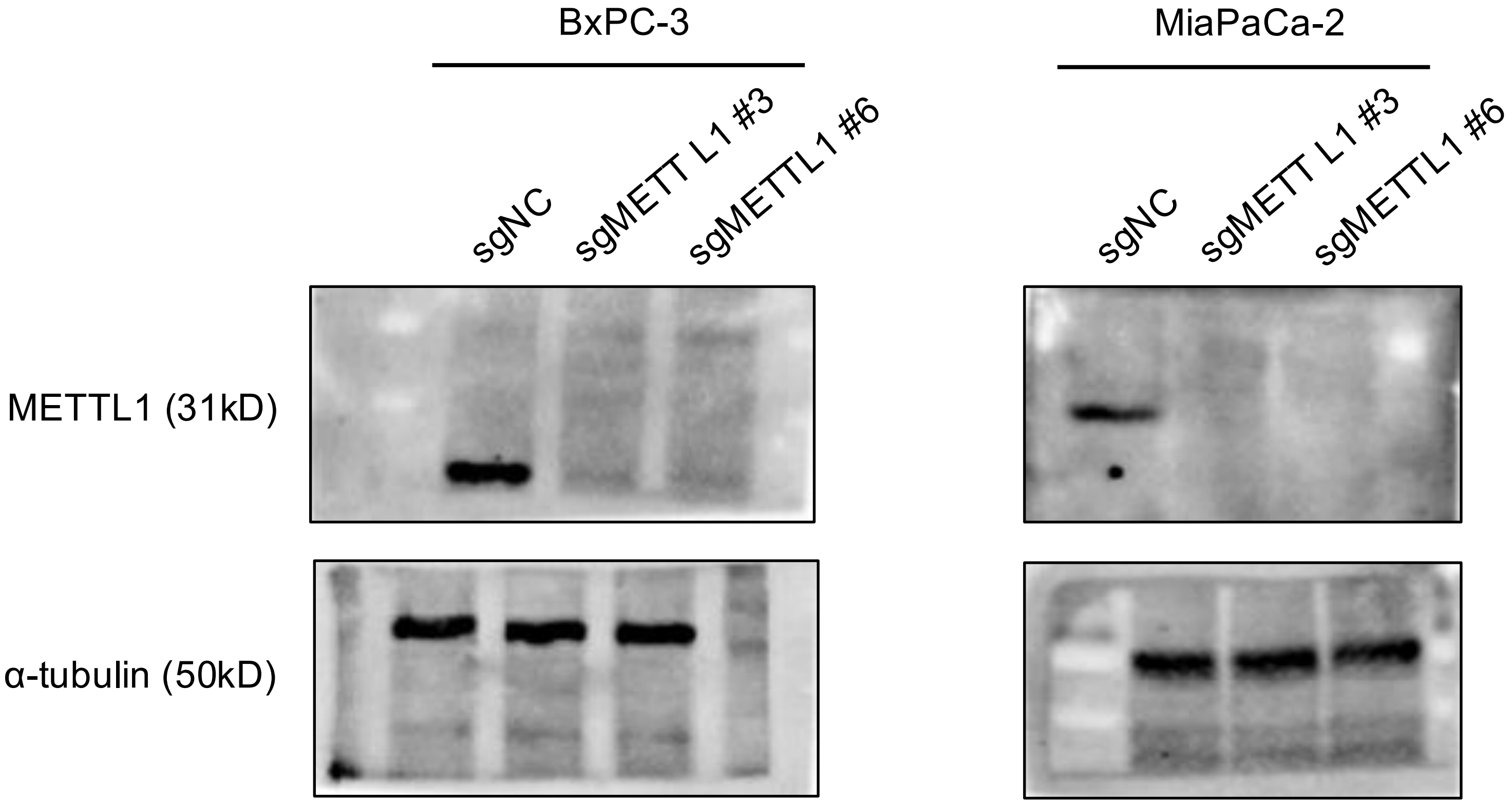

Full unedited blot for Figure 5A

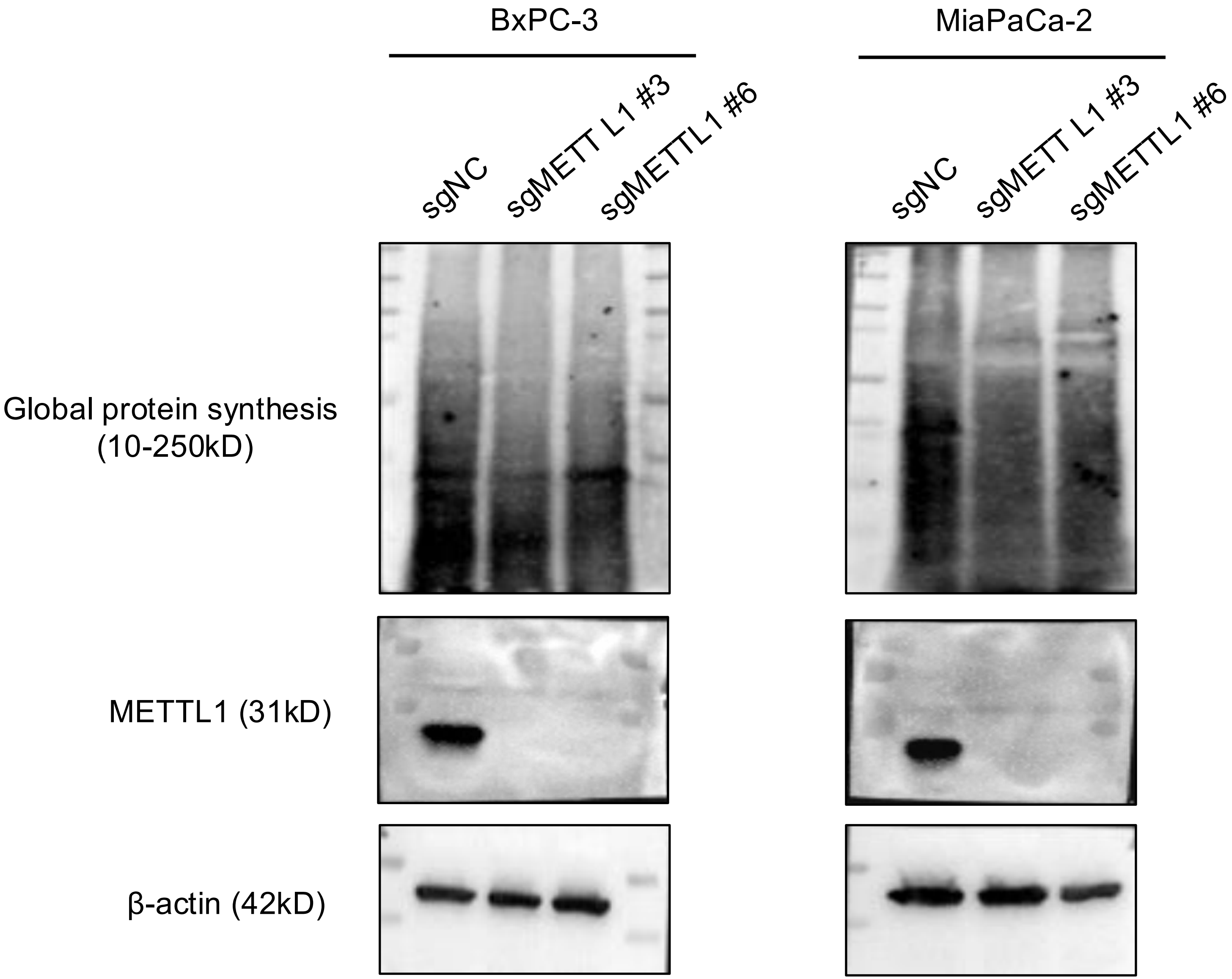

Full unedited blot for Figure 6F

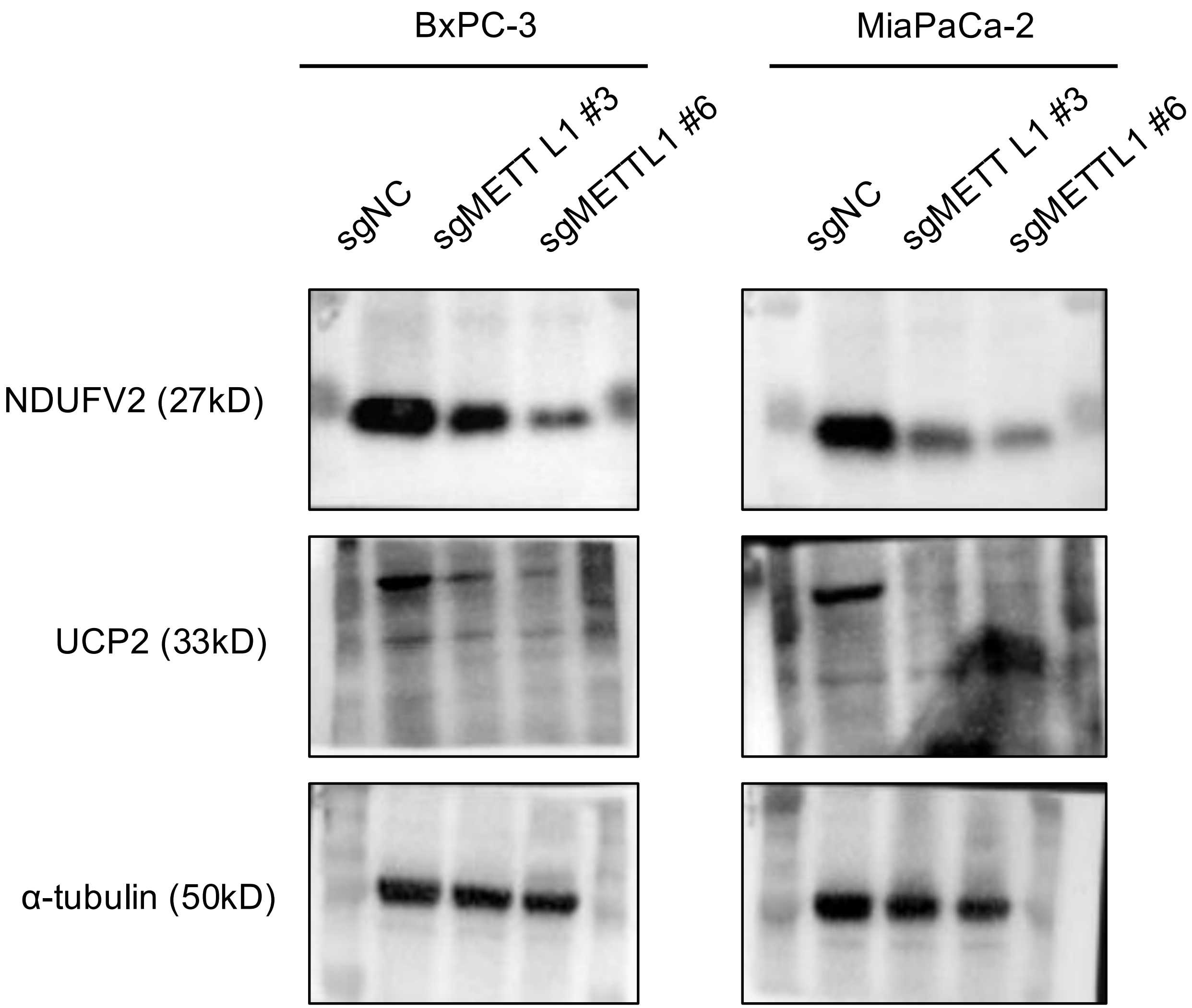

Full unedited blot for Figure 6G

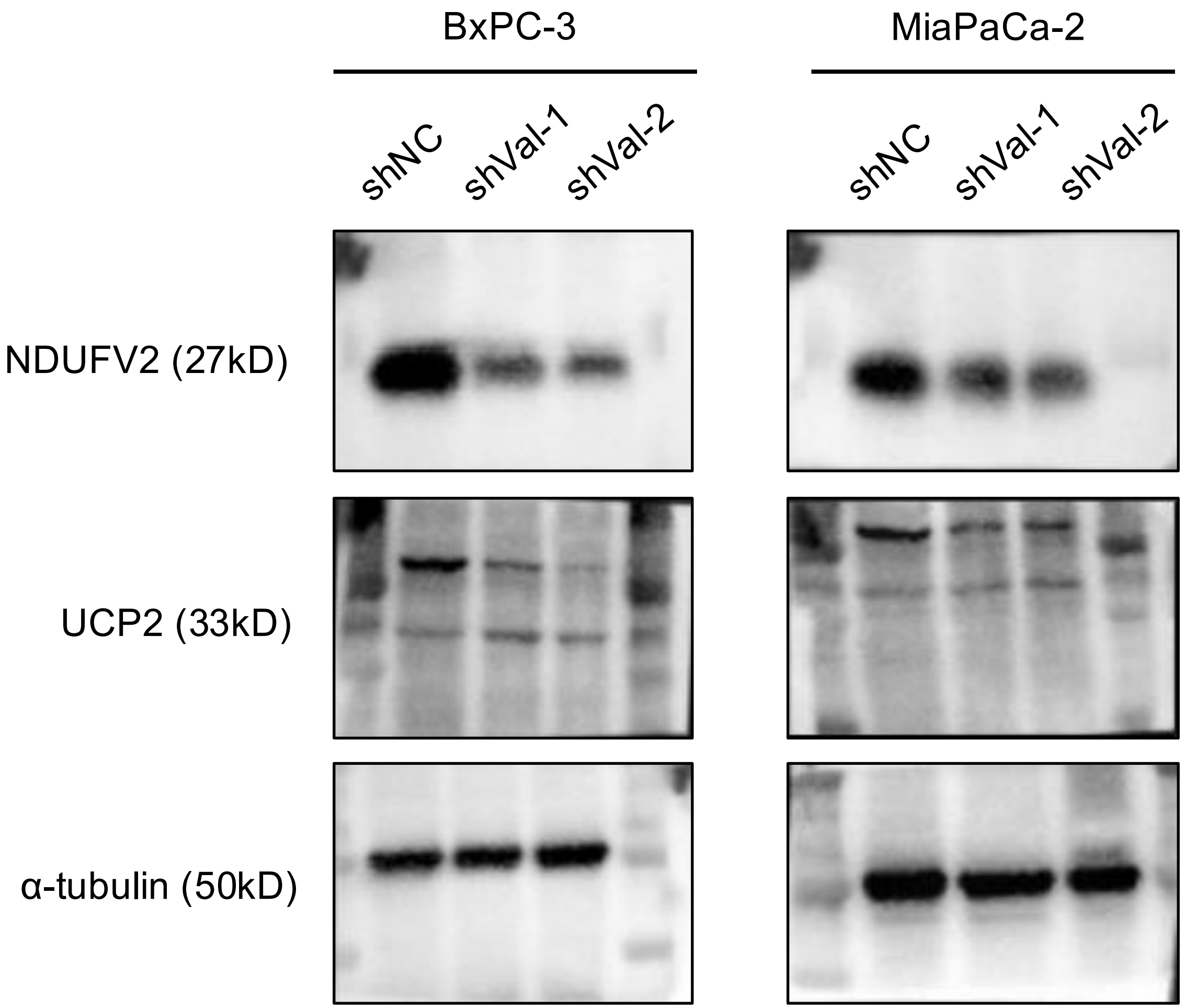

Full unedited blot for Figure S4C

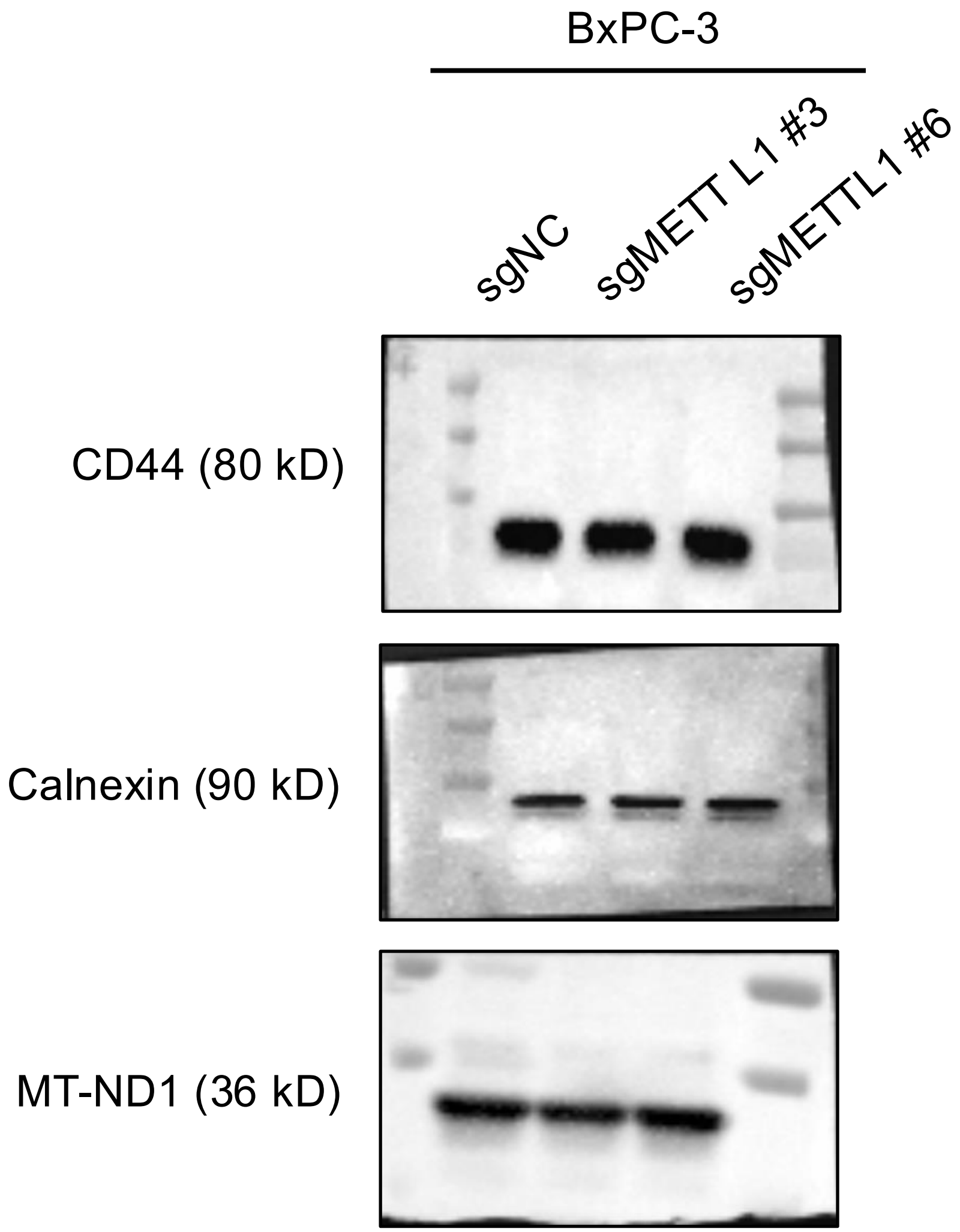

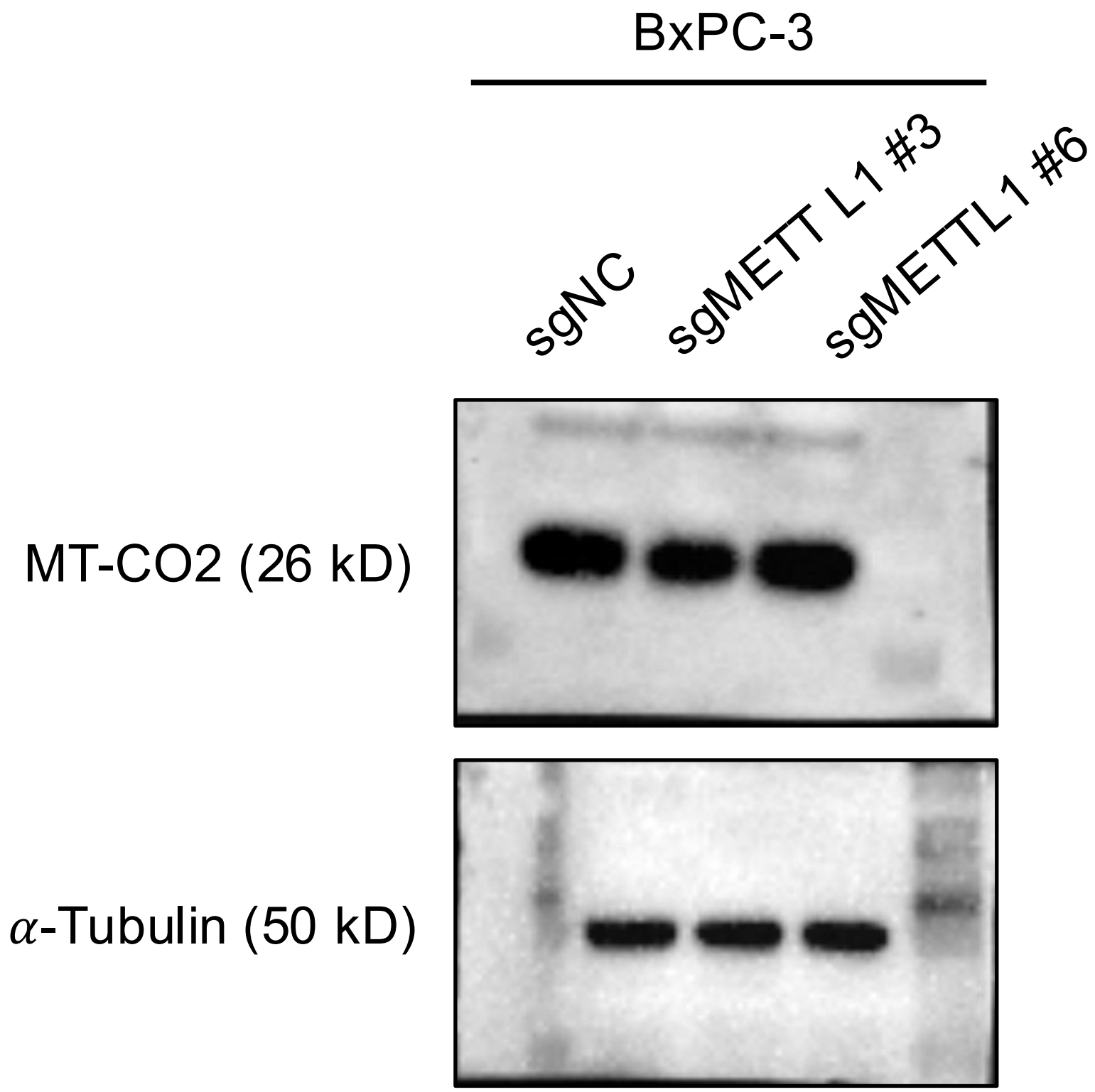

Full unedited blot for Figure S4D

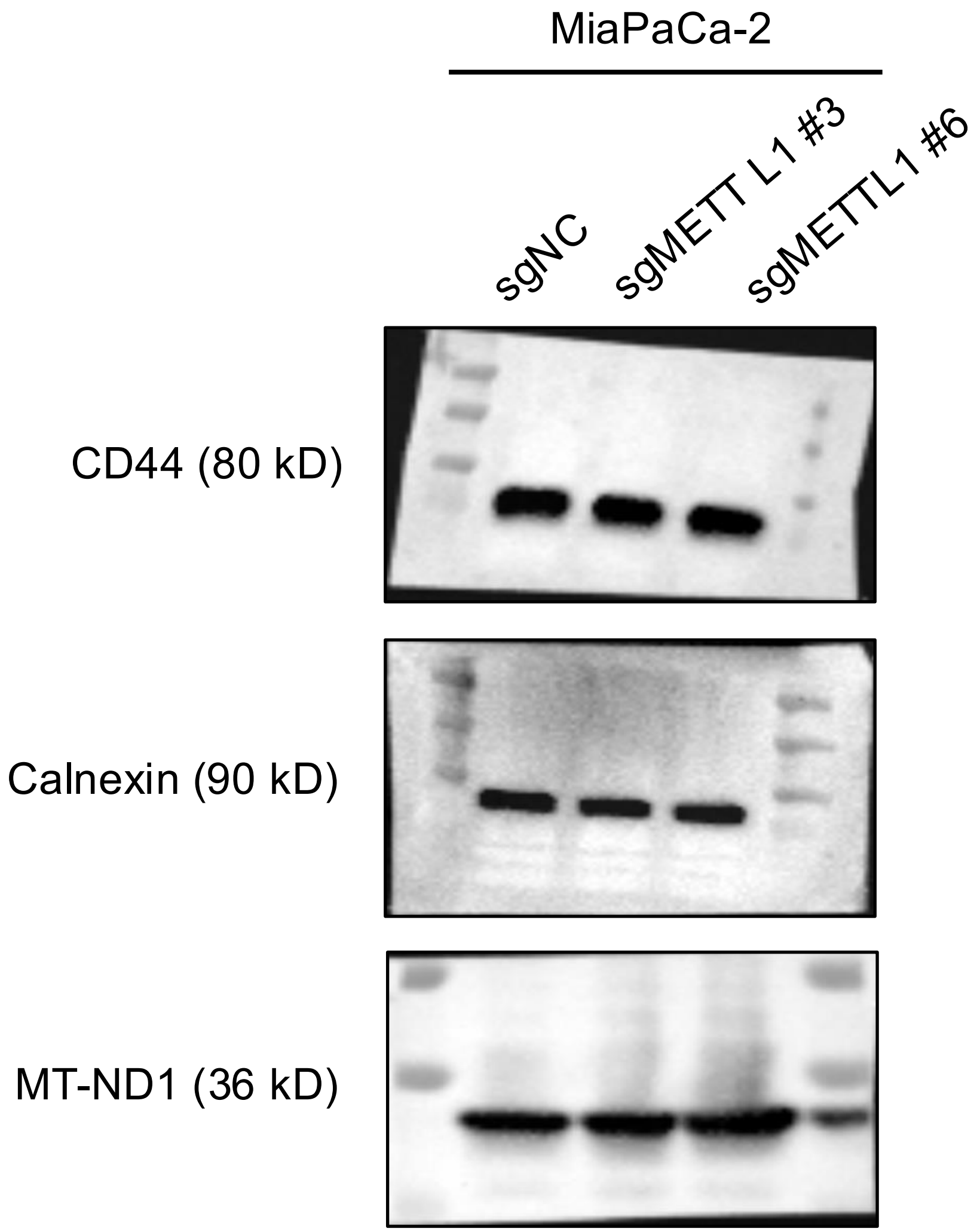

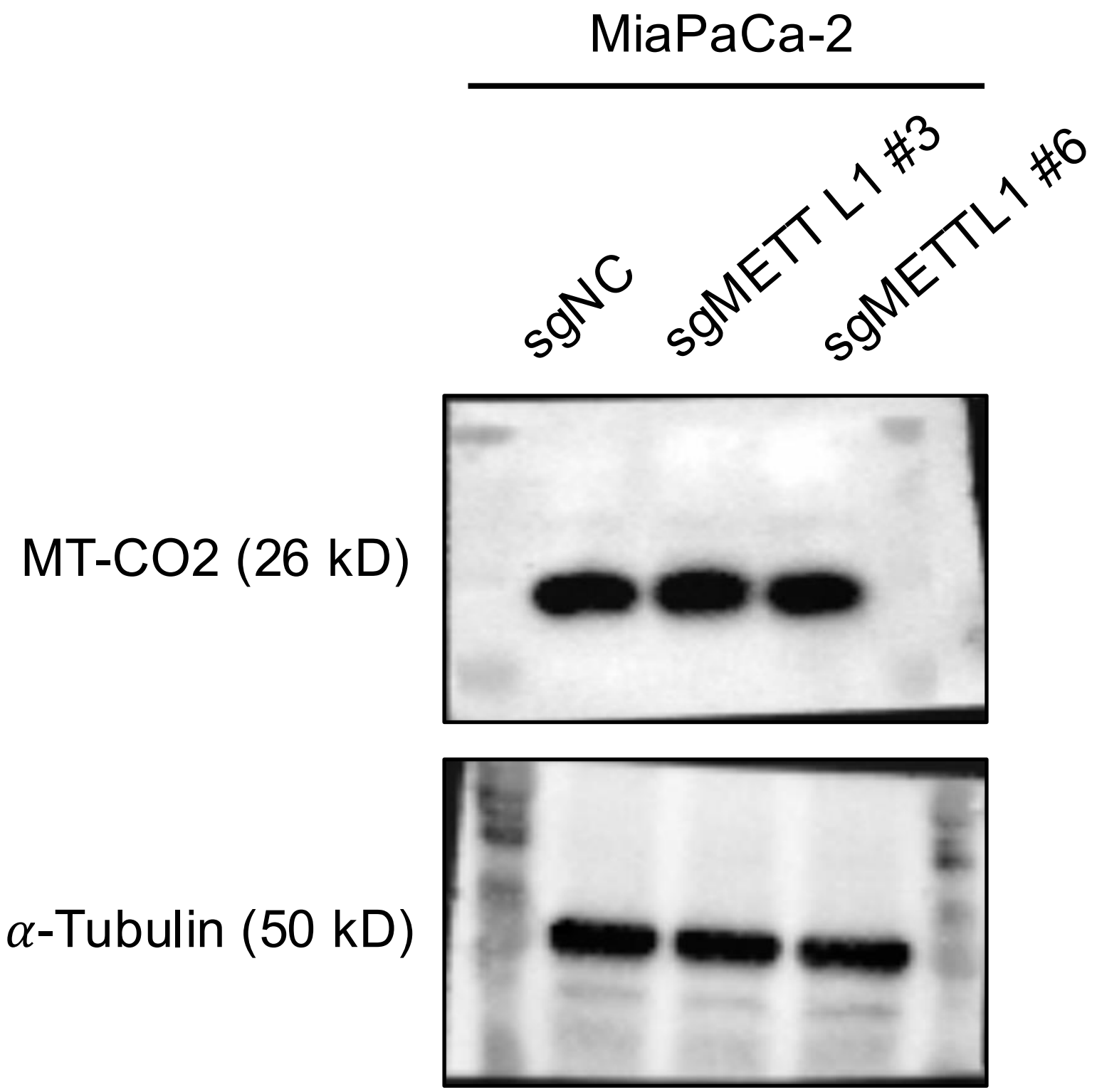

Full unedited blot for Figure S4F

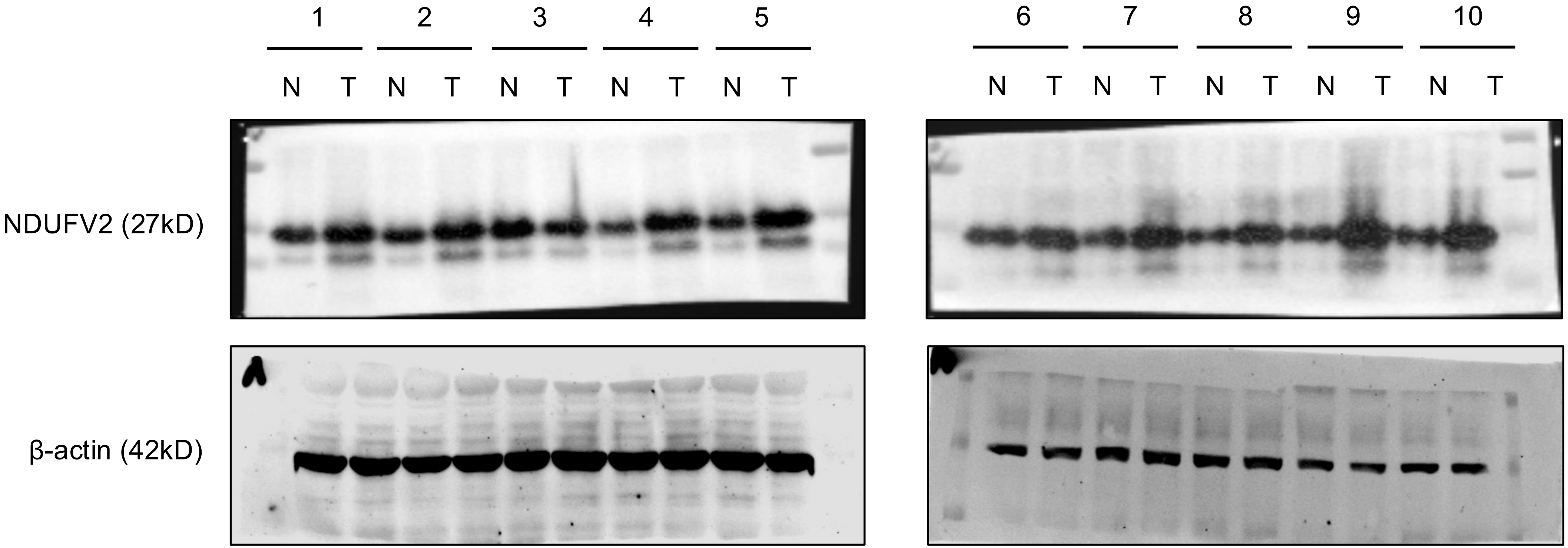

Full unedited blot for Figure S4G

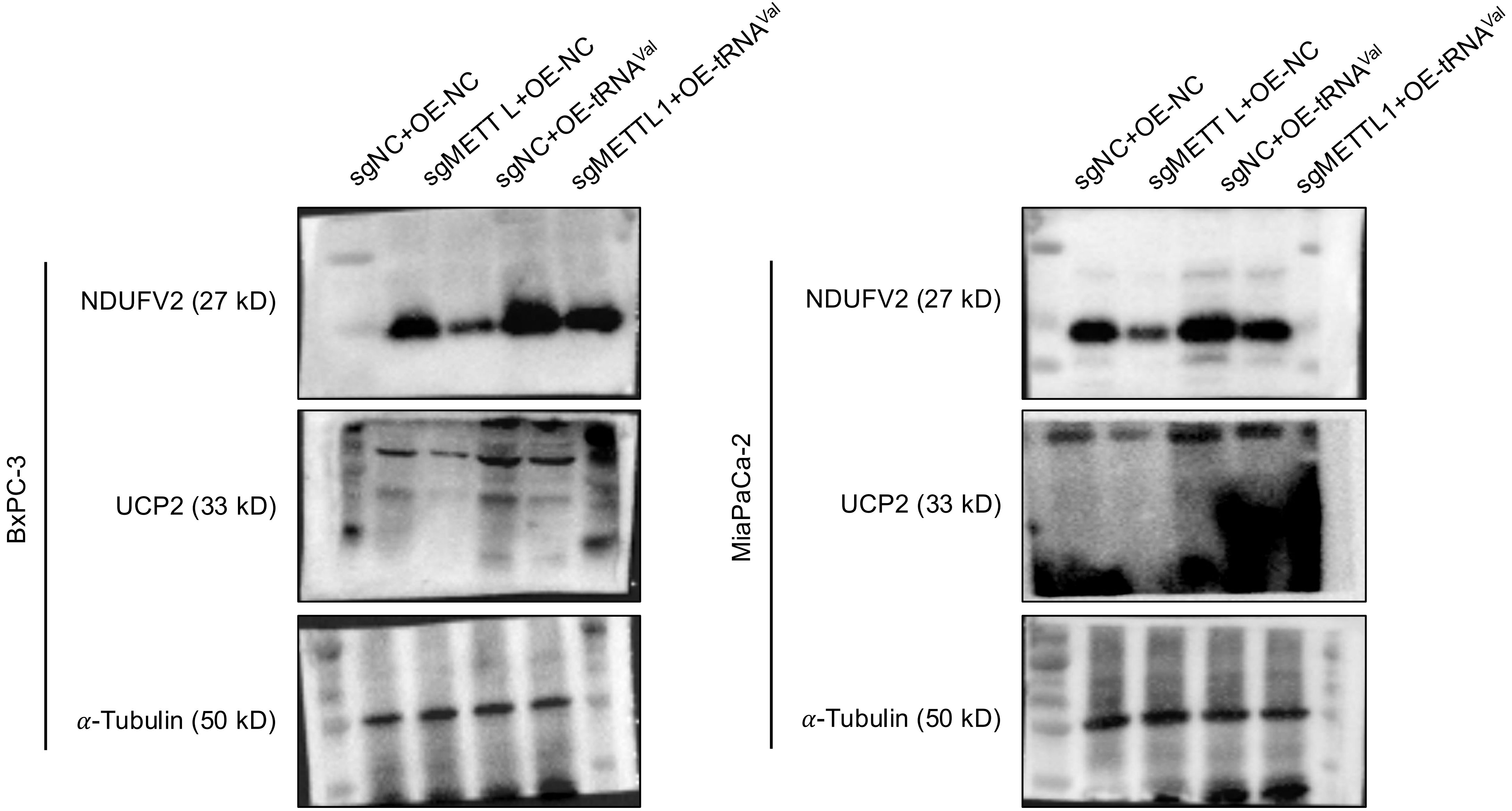

Supplement: Supplementary file 2 [file jci-136-204312-s294.pdf]
